# The Translational Regulators GCN-1 and ABCF-3 Act Together to Promote Apoptosis in *C. elegans*

**DOI:** 10.1371/journal.pgen.1004512

**Published:** 2014-08-07

**Authors:** Takashi Hirose, H. Robert Horvitz

**Affiliations:** Howard Hughes Medical Institute, Department of Biology, Massachusetts Institute of Technology, Cambridge, Massachusetts, United States of America; University of California San Diego, United States of America

## Abstract

The proper regulation of apoptosis requires precise spatial and temporal control of gene expression. While the transcriptional and translational activation of pro-apoptotic genes is known to be crucial to triggering apoptosis, how different mechanisms cooperate to drive apoptosis is largely unexplored. Here we report that pro-apoptotic transcriptional and translational regulators act in distinct pathways to promote programmed cell death. We show that the evolutionarily conserved *C. elegans* translational regulators GCN-1 and ABCF-3 contribute to promoting the deaths of most somatic cells during development. GCN-1 and ABCF-3 are not obviously involved in the physiological germ-cell deaths that occur during oocyte maturation. By striking contrast, these proteins play an essential role in the deaths of germ cells in response to ionizing irradiation. GCN-1 and ABCF-3 are similarly co-expressed in many somatic and germ cells and physically interact *in vivo*, suggesting that GCN-1 and ABCF-3 function as members of a protein complex. GCN-1 and ABCF-3 are required for the basal level of phosphorylation of eukaryotic initiation factor 2α (eIF2α), an evolutionarily conserved regulator of mRNA translation. The *S. cerevisiae* homologs of GCN-1 and ABCF-3, which are known to control eIF2α phosphorylation, can substitute for the worm proteins in promoting somatic cell deaths in *C. elegans*. We conclude that GCN-1 and ABCF-3 likely control translational initiation in *C. elegans*. GCN-1 and ABCF-3 act independently of the anti-apoptotic BCL-2 homolog CED-9 and of transcriptional regulators that upregulate the pro-apoptotic BH3-only gene *egl-1*. Our results suggest that GCN-1 and ABCF-3 function in a pathway distinct from the canonical CED-9-regulated cell-death execution pathway. We propose that the translational regulators GCN-1 and ABCF-3 maternally contribute to general apoptosis in *C. elegans* via a novel pathway and that the function of GCN-1 and ABCF-3 in apoptosis might be evolutionarily conserved.

## Author Summary

Apoptosis, also referred to as programmed cell death, is a crucial cellular process that eliminates unwanted cells during animal development and tissue homeostasis. Abnormal regulation of apoptosis can cause developmental defects and a variety of other human disorders, including cancer, neurodegenerative diseases and autoimmune diseases. Therefore, it is important to identify regulatory mechanisms that control apoptosis. Previous studies have demonstrated that the transcriptional induction of apoptotic genes can be crucial to initiating an apoptotic program. Less is known about translational controls of apoptosis. Here we report that the evolutionarily conserved *C. elegans* translational regulators GCN-1 and ABCF-3 promote apoptosis generally and act independently of the anti-apoptotic BCL-2 homolog CED-9. GCN-1 and ABCF-3 physically interact and maintain the phosphorylation level of eukaryotic initiation factor 2α, suggesting that GCN-1 and ABCF-3 act together to regulate the initiation of translation. We propose that the translational regulators GCN-1 and ABCF-3 maternally contribute to the proper execution of the apoptotic program.

## Introduction

Apoptosis is a naturally occurring process that eliminates unwanted cells during development and maintains tissue homeostasis [1], [2]. For example, apoptosis removes most larval tissues of insects during metamorphosis, sculpts the future inner ear in chicks, eliminates the interdigital web in mammals and shapes the endocardial cushion into valves and septa to generate the four-chamber architecture of the mammalian heart [1], [2]. Apoptosis also culls nearly 80% of oocytes prior to birth in humans and eliminates cells that receive insufficient cell-survival signals to maintain homeostasis [1]. The improper regulation of an apoptotic program can result in either too much or too little cell death, leading to developmental abnormalities and a wide variety of human disorders, such as cancer, neurodegenerative diseases, autoimmune diseases and developmental disorders [3], [4]. It is important to identify mechanisms that regulate apoptosis to understand both animal development and human disorders caused by the dysregulation of apoptosis.

The precise spatial and temporal expression of regulators of apoptosis is known to be crucial for initiating the apoptotic cell-killing program during development and in response to environmental stresses, including ionizing radiation, temperature change, nutrient limitation, oxidative stress and viral infection [1], [2]. Many examples of the transcriptional control of apoptosis have been described. For example, in mammals the genes that encode the pro-apoptotic BCL-2 family member BAX, the BH3-only proteins NOXA, PUMA and BID, the apoptotic protease-activating factor-1 APAF-1 and the death receptor 5 DR5 protein are transcriptionally upregulated by the tumor suppressor p53 transcription factor in response to DNA damage or to the induced expression of p53 [5]–[11], resulting in an induction of apoptosis. The *Drosophila* apoptotic activator gene *reaper* is upregulated by multiple transcriptional regulators, including Hox transcription factors, nuclear hormone receptors, AP-1, Polycomb, p53, and histone-modifying enzymes, to promote the morphogenesis of segment boundaries, metamorphosis, and DNA damage responses [1]. In *C. elegans*, the transcription of the pro-apoptotic BH3-only gene *egl-1* is directly regulated in a cell-specific manner by transcription factors that include the Hox family proteins MAB-5, CEH-20, LIN-39 and CEH-34, the E2F protein EFL-3, the Snail family zinc finger protein CES-1, the Gli family transcription factor TRA-1, and the basic helix-loop-helix proteins HLH-2 and HLH-3 [12]–[15]. The caspase gene *ced-3* is also upregulated by the Hox transcription factor PAL-1 in the tail spike cell before its death [16]. Recently, we showed that the Sp1 transcription factor SPTF-3 directly drives the transcription of both the pro-apoptotic BH3-only gene *egl-1*, which mediates a caspase-dependent apoptotic pathway, and the AMPK-related gene *pig-1*, which mediates a caspase-independent apoptotic pathway [17]. The transcriptional regulation of apoptotic genes clearly plays a crucial role in determining whether specific cells live or die during development.

Translational control is also important for the apoptotic process. In mammals, expression of the pro-apoptotic protein APAF-1 and the anti-apoptotic protein X-chromosome-linked inhibitor of apoptosis XIAP are regulated at the translational level by internal ribosome-entry sites (IRES) [18]. Exposure of cultured mammalian cells to etoposide or UV light induces APAF-1 expression via IRES-mediated translation, resulting in the activation of the caspase-dependent apoptotic program [19]. The protein level of XIAP is increased via IRES-mediated translation under stress conditions, such as serum starvation [20]. However, the specific translational regulators involved in IRES-mediated translation of APAF-1 and XIAP are unknown. In *C. elegans*, the RNA-binding protein GLD-1, which is highly expressed in the transition zone and early pachytene regions of the hermaphrodite gonad, inhibits translation of the mRNA of the p53 homolog *cep-1* by directly binding to the *cep-1* 3′ UTR, thereby preventing *cep-1*-dependent apoptosis in response to DNA damage [21]. Translational initiation factors have also been reported to be involved in the control of apoptosis in *C. elegans*. For example, RNAi knockdown of the *C. elegans* eukaryotic initiation factor-4G IFG-1 induces CED-4 expression in the gonad and increases the frequency of germ-cell death [22], [23]. The eukaryotic initiation factor-3 subunit-k eIF-3.K is partially required for the deaths of somatic cells and acts through the caspase CED-3 to promote those cell deaths [24]. Although many studies have shown that both transcriptional and translational regulation of apoptotic genes is crucial for controlling apoptotic programs, how transcriptional and translational mechanisms are coordinated to promote apoptosis remains elusive.

Here we show that the maternally-contributed translational regulators GCN-1 and ABCF-3 act together to promote the cell deaths of possibly all somatic cells and of germ cells in response to ionizing radiation in a pathway distinct from the BCL-2 homolog CED-9-regulated canonical cell-death execution pathway of *C. elegans*. GCN-1 and ABCF-3 are required to maintain the basal level of phosphorylation of eukaryotic initiation factor 2 (eIF2α). The functions of GCN-1 and ABCF-3 in the promotion of programmed cell death are evolutionarily conserved between *C. elegans* and *Saccharomyces cerevisiae*. We show that GCN-1 and ABCF-3 cooperate with the transcriptional regulators CEH-34, EYA-1 and SPTF-3 and the protein kinase PIG-1 to promote the death of a specific somatic cell, the sister cell of the pharyngeal M4 motor neuron. We propose that the evolutionarily-conserved translational regulators GCN-1 and ABCF-3 contribute to apoptosis in general.

## Results

### The translational regulators GCN-1 and ABCF-3 are required for M4 sister cell death

**Figure 1:**
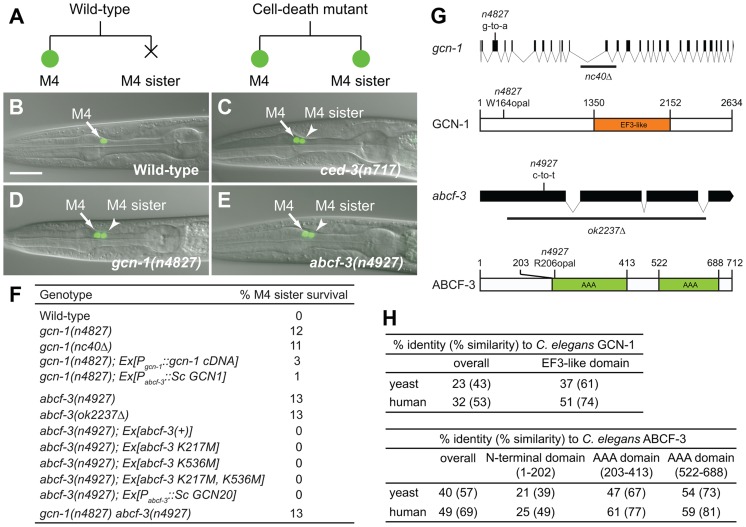
*gcn-1(n4827)* and *abcf-3(n4927)* cause a defect in M4 sister cell death. (A) Schematic representation of the M4 cell lineage in the wild type and mutants defective in M4 sister cell death. X, programmed cell death. (B–E) Merged epifluorescence and Nomarski images of the pharynx in wild-type, *ced-3(n717)*, *gcn-1(n4827)* and *abcf-3(n4927)* animals expressing *P_ceh-28_::gfp*. Arrow, M4 neuron. Arrowhead, surviving M4 sister. Scale bar, 20 µm. Panels B and C from ref. 15. (F) The percentages of M4 sister survival in animals of the indicated genotypes. (G) Genomic organizations and protein structures of *gcn-1* and *abcf-3*, including the locations and natures of the mutations *n4827* and *n4927*. Orange box, EF3-like domain. Green boxes, AAA domains. Black bars, sequences deleted in alleles *gcn-1(nc40Δ)* and *abcf-3(ok2237Δ)*. (H) Comparison of amino acid sequences of the entire protein or the EF3-like domain of GCN-1 and of the entire protein, the N-terminal domain, the first AAA domain or the second AAA domain of ABCF-3 among yeast, *C. elegans* and humans. Clustal W was used to align amino-acid sequences and to calculate identity and similarity.

The *C. elegans* pharyngeal M4 motor neuron is generated during embryonic development and survives to regulate pharyngeal muscle contraction in feeding behavior, whereas the M4 sister cell dies by programmed cell death soon after its generation (Figure 1A) [25], [26]. We created a *P_ceh-28_::gfp* reporter transgene that expresses GFP specifically in the M4 neuron of wild-type animals and in both the M4 neuron and the surviving M4 sister of *ced-3* caspase mutants defective in programmed cell death (Figure 1B–1C). This reporter allowed us to easily identify mutants with a defect in M4 sister cell death. [15]. Using this reporter, we performed a genetic screen for mutations that cause a defect in M4 sister cell death. Among our isolates were two non-allelic mutations, *n4827* and *n4927*, that caused M4 sister survival in 12% of *n4827* mutants and 13% of *n4927* mutants (Figure 1D–1F).

We mapped *n4827* to a 175 kb interval of chromosome III containing 18 predicted genes (Figure S1A). We used whole-genome sequencing to identify four strain-specific unique homozygous mutations within this interval in *n4827* animals (Figure S1A) [27]. Of the four mutations, only one was exonic. This mutation was located in the third exon of *gcn-1*, which encodes a homolog of the *S. cerevisiae* Gcn1p protein. The *n4827* mutation is predicted to change the tryptophan 164 codon to an opal stop codon, generating a small truncated protein (Figure 1G). A deletion mutation of *gcn-1*, *nc40Δ*, phenocopied the *n4827* mutation [28]: 11% of *gcn-1(nc40Δ)* mutants and 12% of *n4827* mutants had a surviving M4 sister, respectively (Figure 1F). The cell-death defect of *n4827* mutants was partially rescued by a transgene that express *gcn-1* cDNA under the control of the *gcn-1* promoter (Figure 1F). These results indicate that *n4827* is likely a null allele of *gcn-1* and that loss of *gcn-1* function causes a defect in M4 sister cell death.

We mapped *n4927* to a 5.3 Mb interval of chromosome III (Figure S1B). This interval contains the gene *abcf-3*, which encodes a homolog of the *S. cerevisiae* Gcn20p protein [29]. Gcn20p physically interacts with Gcn1p, the *S. cerevisiae* homolog of GCN-1 [30]. We determined the sequence of *abcf-3* in *n4927* animals and identified a mutation that changes the arginine 206 codon to an opal stop codon (Figure 1G). A deletion mutation of *abcf-3*, *ok2237Δ*, that removes most of the *abcf-3* coding region phenocopied the *n4927* mutation: 13% of *abcf-3(ok2237Δ)* mutants and 13% of *n4927* mutants had a surviving M4 sister (Figure 1F). Furthermore, the cell-death defect of *n4927* mutants was completely rescued by a transgene carrying only the *abcf-3* genomic locus. We concluded that *n4927* is likely a null allele of *abcf-3* and that loss of *abcf-3* function causes a defect in M4 sister cell death.

*abcf-3* encodes an AAA ATPase protein with two AAA domains (Figure 1G). In many proteins AAA domains have ATPase activity. To determine whether ATPase activity is important for ABCF-3 to promote M4 sister cell death, we generated *abcf-3* transgenes carrying mutations that presumably inactivate the ATPase activity of each AAA domain by altering the lysine residues known to be catalytically essential for other AAA ATPases [31]. A wild-type *abcf-3* transgene as well as mutant *abcf-3* transgenes that changed lysine 217 of the first AAA domain to methionine [*abcf-3 (K217M)*], lysine 536 of the second AAA domain to methionine [*abcf-3 (K536M*)] or both lysine residues [*abcf-3 (K217M, K536M)*] completely rescued the defect in M4 sister cell death of *abcf-3(n4927)* mutants (Figure 1F). These results support the idea that the ATPase activity of ABCF-3 is dispensable for M4 sister cell death. This result is consistent with studies of *S. cerevisiae* Gcn20p, the homolog of *C. elegans* ABCF-3. Gcn20p that lacks the ATPase activities of both AAA domains because of mutations in conserved glycine residues (Gly371 and Gly654) or because of the deletion of two AAA domains still retains Gcn20p function comparable to that of wild-type Gcn20p [32].

### GCN-1 and ABCF-3 act together to promote M4 sister cell death

GCN-1 and ABCF-3 are evolutionarily conserved among *S. cerevisiae*, *C. elegans* and humans (Figure 1H, Figure S2 and S3). Expression of *S. cerevisiae GCN1*, the homolog of *C. elegans gcn-1*, and *GCN20*, the homolog of *C. elegans abcf-3*, under the control of the *abcf-3* promoter rescued the defect in M4 sister cell death of *C. elegans gcn-1* and *abcf-3* mutants, respectively, indicating that *S. cerevisiae GCN1* and *GCN20* are functional homologs of *C. elegans gcn-1* and *abcf-3*, respectively (Figure 1F).

**Figure 2:**
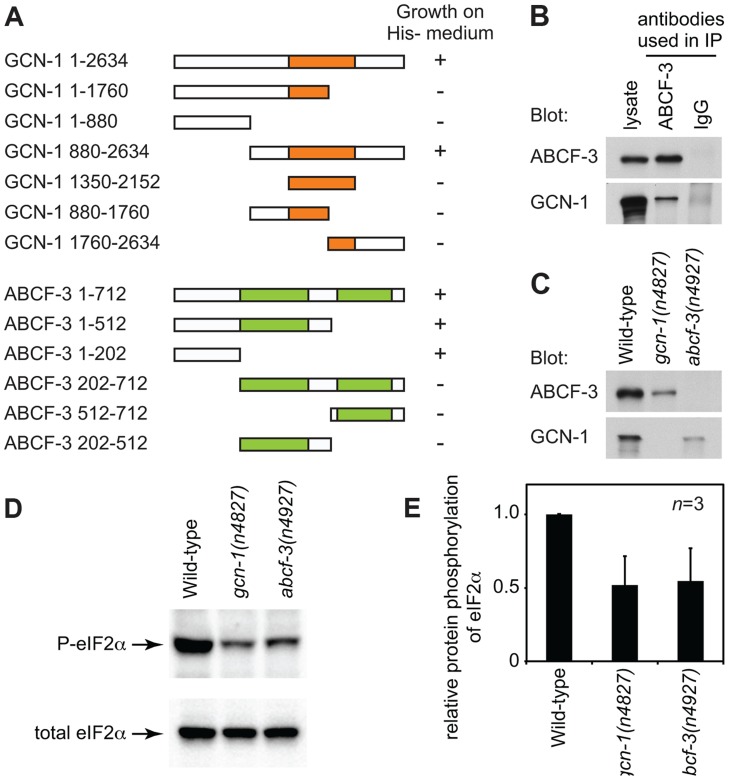
GCN-1 and ABCF-3 proteins are evolutionarily conserved functionally. (A) Schematic representations of the GCN-1 and ABCF-3 proteins used in the yeast two-hybrid binding assays. Orange box, EF3-like domain. Green boxes, AAA domains. The growth of yeast on histidine-minus medium is summarized. +, wild-type growth. −, no or little growth. (B) Western blot analysis of immunocomplexes purified from wild-type animals with an anti-ABCF-3 antibody or control IgG. Both ABCF-3 and GCN-1 are present in the immunocomplex purified with an anti-ABCF-3 antibody but not with the control IgG. (C) Western blot analysis showing levels of ABCF-3 and GCN-1 proteins in fourth-larval stage animals of the indicated genotypes. (D) Western blot analysis showing the levels of eIF2α with phosphorylated serine 49 and total eIF2α of fourth-larval stage animals of the indicated genotypes. (E) Relative intensities of phosphorylated eIF2 to total eIF2 of fourth-larval stage animals of the indicated genotypes. Errors, standard deviations.

*S. cerevisiae* Gcn1p has a domain (amino acids 1350–2152) similar to that of translation elongation factor 3 (EF3). The EF3-like domain is highly conserved among species (Figure 1H and Figure S2) and is necessary and sufficient for binding to Gcn20p [32]. We therefore tested whether *C. elegans* GCN-1 can physically interact with ABCF-3 using the yeast two-hybrid assay (Figure 2A). Full-length GCN-1 (1–2634) interacted with full-length ABCF-3 (1–712). To identify the protein domains important for GCN-1 to bind to ABCF-3, we generated a series of deletion constructs of GCN-1 and assayed each for ABCF-3-binding activity using the yeast two-hybrid assay. GCN-1 fragments not containing entire the EF3 domain (1–1760, 1–880, 880–1760 and 1760–2634) or containing only the EF3 domain (1350–2150) failed to bind ABCF-3, whereas GCN-1 fragments containing the EF-3 domain and surrounding regions (880–2634) bound ABCF-3. These results suggest that GCN-1 physically interacts with ABCF-3 but that unlike in yeast the EF3-like domain is not sufficient for GCN-1 to bind to ABCF-3.

We also defined the domains of ABCF-3 important for ABCF-3 to bind to GCN-1 (Figure 2A). ABCF-3 fragments lacking the N-terminal region (202–712, 512–712 and 202–512) failed to bind GCN-1, whereas ABCF-3 fragments containing the N-terminal region (1–712, 1–512 and 1–202) bound GCN-1, suggesting that the N-terminal portion of ABCF-3 (which does not include the first AAA domain) is necessary and sufficient for binding to GCN-1, just as the N-terminal region of *S. cerevisiae* Gcn20p is necessary and sufficient for binding to Gcn1p, the *S. cerevisiae* homolog of GCN-1.

To determine whether GCN-1 and ABCF-3 interact *in vivo*, we generated antibodies against GCN-1 and ABCF-3 and performed co-immunoprecipitation experiments. We first tested whether these antibodies specifically recognize GCN-1 or ABCF-3 protein using western blot analysis. The antibodies against GCN-1 or ABCF-3 recognized proteins of the sizes predicted for the GCN-1 or ABCF-3 proteins in wild-type animals but not in *gcn-1(n4827)* or *abcf-3(n4927)* animals, respectively, confirming the specificity of these antibodies (Figure 2C). Then we tested whether GCN-1 could be co-immunoprecipitated with ABCF-3. Whole-protein extracts from wild-type animals were subjected to immunoprecipitation using an anti-ABCF-3 antibody (or normal IgG as a control), and then immunocomplexes were analyzed by western blotting using antibodies against ABCF-3 or GCN-1. Both ABCF-3 and GCN-1 were recovered in an immunocomplex purified with the anti-ABCF-3 antibody, whereas neither ABCF-3 nor GCN-1 was recovered in an immunocomplex purified with normal IgG (Figure 2B). We conclude that GCN-1 and ABCF-3 are present in the same protein complex *in vivo*.

Since GCN-1 and ABCF-3 form a complex *in vivo*, we suspected that deletion of either protein might affect the stability of the other protein [29], [33]. To test this hypothesis, we examined the levels of GCN-1 and ABCF-3 proteins by western blot analyses of whole-protein extracts prepared from wild-type, *gcn-1(n4827)* and *abcf-3(n4927)* animals using antibodies against ABCF-3 or GCN-1. The steady-state level of ABCF-3 protein was decreased in *gcn-1(n4827)* animals by 3.6 fold compared to that of wild-type animals. Similarly, the steady-state level of GCN-1 protein was decreased in *abcf-3(n4927)* animals by 4.4 fold (Figure 2C). These results suggest that a lack of ABCF-3 or GCN-1 protein affects the stability of the other protein and support our conclusion that GCN-1 and ABCF-3 are in a protein complex together *in vivo*.

If GCN-1 and ABCF-3 physically interact *in vivo* to promote M4 sister cell death, GCN-1 and ABCF-3 should act together in the same pathway. Since *gcn-1(n4827)* and *abcf-3(n4927)* are likely null mutations, the *gcn-1(n4827)* mutation would not enhance the M4 sister cell-death defect of *abcf-3(n4927)* mutants if *gcn-1* and *abcf-3* function in the same process or pathway. Indeed, we observed no enhancement of the M4 sister cell-death defect of *gcn-1(n4827) abcf-3(n4927)* double mutants compared to that of either single mutant: there was 13% M4 sister survival in *gcn-1(n4827) abcf-3(n4927)* double mutants, 12% M4 sister survival in *gcn-1(n4827)* mutants and 13% M4 sister survival in *abcf-3(n4927)* mutants (Figure 1F). We conclude that *gcn-1* and *abcf-3* function together in the same process of pathway to promote M4 sister cell death, consistent with our finding that GCN-1 and ABCF-3 physically interact *in vivo*.

In *S. cerevisiae*, Gcn1p and Gcn20p are required for the efficient phosphorylation of eukaryotic initiation factor 2 (eIF2α) under both normal conditions and conditions of amino-acid starvation [29], [30]. Gcn1p and Gcn20p form a protein complex that activates the serine-threonine protein kinase Gcn2p, which then phosphorylates an evolutionarily conserved serine residue of eIF2α. The amino acid sequences surrounding the eIF2α phosphorylation site are identical in *S. cerevisiae*, *C. elegans* and humans, suggesting a conserved regulatory mechanism of eIF2α [28]. We tested whether GCN-1 and ABCF-3 promote the phosphorylation of eIF2α in *C. elegans* using an antibody that specifically recognizes eIF2α that is phosphorylated at serine 49 (P-eIF2α). From wild-type animals cultivated under normal physiological conditions, a single band of eIF2α was detected in western blotting analyses using either the anti-P-eIF2α antibody or an antibody that recognized total eIF2α (Figure 2D). In *gcn-1(n4827)* and *abcf-3(n4927)* mutants, the phosphorylation levels of eIF2α in physiological conditions were 52% and 54% of the levels in wild-type animals, respectively (Figure 2D and 2E). We conclude that *gcn-1* and *abcf-3* are required to maintain the steady-state level of the phosphorylation of eIF2α.

**Figure 3:**
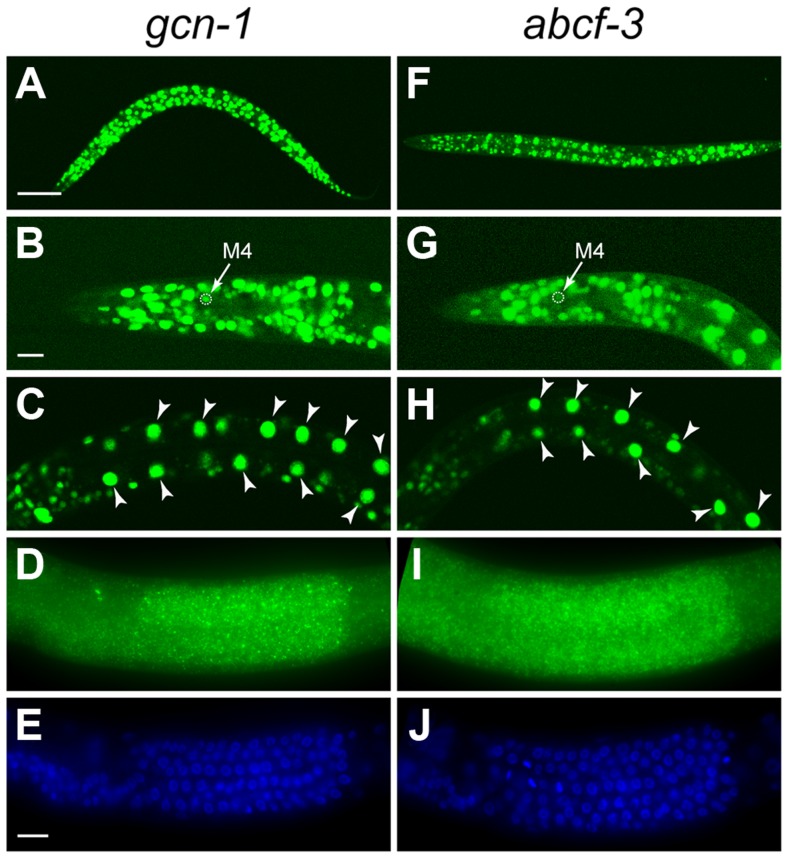
*gcn-1* and *abcf-3* are ubiquitously expressed. (A–E) Expression pattern of *gcn-1*. (A–C) Expression of GFP under the control of the *gcn-1* promoter in second-larval stage animals and (D and E) FISH of *gcn-1* mRNA in third-larval stage animals. Green, *gcn-1* mRNA. Blue, DAPI staining of nuclei. (F–J) Expression pattern of *abcf-3*. (F–H) Expression of GFP under the control of the *abcf-3* promoter in the second-larval stage animals and (I and J) FISH of *abcf-3* mRNA in third-larval stage animals. Green, *abcf-1* mRNA. Blue, DAPI staining of nuclei. Arrows, M4 neuron. Arrowheads, intestinal cells. Scale bars, 50 µm in A and F; 10 µm in B, C, G, H; and 5 µm in D, E, I and J.

The regulation of phosphorylation of eIF2α plays an essential role in the initiation of translation. We therefore directly tested whether *gcn-1* and *abcf-3* affect gene expression at the translational level. Since *gcn-1* and *abcf-3* are highly expressed in the gonads at the fourth larval stage, maternally contribute to the death of the M4 sister and affect most programmed cell deaths (see below, Figure 3D and 3I, Table 1 and Table S5), we isolated both wild-type animals and *gcn-1* and *abcf-3* mutants at the fourth larval stage and performed mRNA-seq and ribosome profiling (Ribo-seq) to generate quantitative genome-wide information concerning mRNA abundance and the locations of mRNAs occupied by ribosomes [34]. Parallel analyses of data from Ribo-seq and mRNA-seq studies allowed us to distinguish differences in mRNA abundance from differences in translational control and to generate a quantitative and comprehensive list of genes the expression of which is likely regulated by *gcn-1* and *abcf-3* at the translational level. Loss of *gcn-1* or *abcf-3* function affected the expression of a large number of genes at either the transcriptional or translational level or at both (Figure S4A and S4B and Table S1). Since GCN-1 and ABCF-3 very likely function in translational control, their effects on transcript levels are likely indirect. Changes in gene expression compared to wild-type animals were similar between *gcn-1* and *abcf-3* mutants, supporting our conclusion that *gcn-1* and *abcf-3* act together (Figure S4C and S4D). The expression of 464 genes or 217 genes changed in both *gcn-1* and *abcf-3* mutants compared to wild-type animals at least two-fold (*p*<0.1) in mRNA-seq or Ribo-seq analyses, respectively (Figure S4E and S4F). Of the 217 genes altered in translational expression, 98 genes showed no alterations in mRNA levels using our standards of a two-fold change and *p*<0.1 (Table S2 and Table S3). These genes are candidates for being directly regulated by both *gcn-1* and *abcf-3* translationally. These results suggest that *gcn-1* and *abcf-3* function together in the translational control of many genes.

**Table 1:** *gcn-1(n4827)* and *abcf-3(n4927)* enhance the cell-death defects of partial loss-of-function *ced-3(n2427)* mutants.

Genotype	M4 sister	NSM sisters	PVQ sisters	g1A sisters	RIM and RIC sisters	Extra cells±SD (anterior pharynx)
Wild-type	0	0	0	0	0	0.0±0.0
*gcn-1(n4827)*	12	0	1^1^	1^1^	0^1^	0.1±0.3
*abcf-3(n4927)*	13	0	1^2^	1^2^	0^2^	0.1±0.3
*ced-3(n2427)*	48	16	4	13	23	1.4±0.9
*gcn-1(n4827); ced-3(n2427)*	98	38	16^1^	36^1^	44	3.7±1.1^3^
*abcf-3(n4927); ced-3(n2427)*	94	34	14^2^	45^2^	46	3.8±1.3^3^

The percentages of the survival of the M4 sister, NSM sisters, PVQ sisters, g1A sisters and RIM and RIC sisters or numbers of extra cells in the anterior pharynx.

More than 100 cells were scored for survival of the M4 sister, NSM sisters, PVQ sisters, g1A sisters and RIM and RIC sisters.

24 animals were scored for extra cells in the anterior pharynx.

All strains were homozygous for the reporter transgene to score survival of the indicated cells except for the extra cells in the anterior pharynx. See Materials and Methods for the reporter transgenes.

1 These strains were homozygous for *unc-45(r450)*.

2 These strains were homozygous for *dpy-18(e364)*.

3 Student's *t*-test compared with *ced-3(n2427)*, *P*<0.0001.

In mammals, eIF2α phosphorylation is mediated by at least four different protein kinases: PKR-like endoplasmic reticulum kinase (PERK), general control non-derepresessible-2 (GCN2), double-stranded RNA-activated protein kinase (PKR) and heme-regulated inhibitor kinase (HRI); each of these kinases is activated by a distinct stress signal [18]. These kinases share homology in their kinase catalytic domains, but their effector domains are distinct and are subject to different regulatory mechanisms. Homologs of genes encoding two of these protein kinases exist in the *C. elegans* genome: the PERK homolog PEK-1 and the GCN2 homolog GCN-2. Y38E10A.8 has a kinase domain similar to that of mammalian eIF2α kinases but does not have an obvious homolog. We tested whether these three protein kinases are required for the programmed cell death of the M4 sister. Neither single mutants of each kinase gene nor the triple mutant was defective in M4 sister cell death (Table S4), suggesting that one or more unidentified protein kinase(s) regulated by GCN-1 and ABCF-3 are responsible for phosphorylating eIF2α in the regulation of M4 sister cell death. Alternatively, it is possible GCN-1 and ABCF-3 promote M4 sister cell death through one or more targets other than eIF2α.

### *gcn-1* and *abcf-3* are expressed ubiquitously

To determine the expression patterns of *gcn-1* and *abcf-3*, we generated transgenes expressing a reporter GFP under the control of the endogenous *gcn-1* or *abcf-3* promoter. Both *gcn-1* and *abcf-3* were expressed in most cells during all stages of development. We observed *gcn-1* and *abcf-3* expression in head neurons, hypodermal cells, intestinal cells, body wall muscles, and pharyngeal neurons, including the M4 neuron (Figure 3A–3C and 3F–3H). We also used the technique of fluorescence *in situ* hybridization (FISH) with a level of sensitivity sufficient to detect single mRNA molecules [35] to observe endogenous *gcn-1* and *abcf-3* transcripts. Consistent with the expression of the GFP reporter transgenes, *gcn-1* and *abcf-3* mRNAs were observed in most somatic cells. In addition, *gcn-1* and *abcf-3* mRNAs were abundant in the germ cells in the hermaphrodite gonad (Figure 3D, 3E, 3I and 3J). The similar expression patterns of *gcn-1* and *abcf-3* are consistent with our observations that GCN-1 and ABCF-3 physically interact and act together to promote the death of the M4 sister.

Since *gcn-1* and *abcf-3* are ubiquitously expressed and required to broadly maintain the basal level of phosphorylation of eIF2α, we tested whether *gcn-1* and *abcf-3* might be involved in other biological processes. We did not observe abnormalities in the morphologies of the hermaphrodite vulva, the male tail or the neurite processes of the M4, I2 and PVQ neurons. However, the growth rate of *gcn-1* and *abcf-3* mutants from embryogenesis to the fourth larval stage was around 24 hours longer than that of wild-type animals, and the mitotic pachytene region of the hermaphrodite gonad was expanded over the loop regions of the gonads. (data not shown). These observations suggest that *gcn-1* and *abcf-3* affect biological processes in addition to programmed cell death.

### GCN-1 and ABCF-3 promote the deaths of most somatic cells during development and of germ cells in response to ionizing radiation

Given the ubiquitous expression patterns of *gcn-1* and *abcf-3*, we tested whether *gcn-1* and *abcf-3* promote programmed cell deaths in addition to that of the M4 sister. We examined *gcn-1(n4827)* and *abcf-3(n4927)* mutants for defects in the deaths of the NSM sisters, the PVQ sisters, the g1A sisters, the RIM and RIC sisters and multiple cells in the anterior pharynx. *gcn-1(n4827)* and *abcf-3(n4927)* single mutants did not exhibit defects in the deaths of these cells (Table 1). However, when either the *gcn-1(n4827)* or the *abcf-3(n4927)* mutation was combined with the partial loss-of-function *ced-3(n2427)* mutation, which sensitizes strains to weak defects in cell death [36], we observed significant cell-death defects for all cell types tested (Table 1). For example, the *gcn-1(n4827)* and *abcf-3(n4927)* mutations enhanced the *ced-3(n2427)* defect from 16% to 38% and 34%, respectively, for the NSM sister and from 13% to 36% and 45%, respectively, for the g1A sister. We conclude that *gcn-1* and *abcf-3* promote programmed cell death generally rather than specifically affecting the M4 sister cell death.

**Figure 4:**
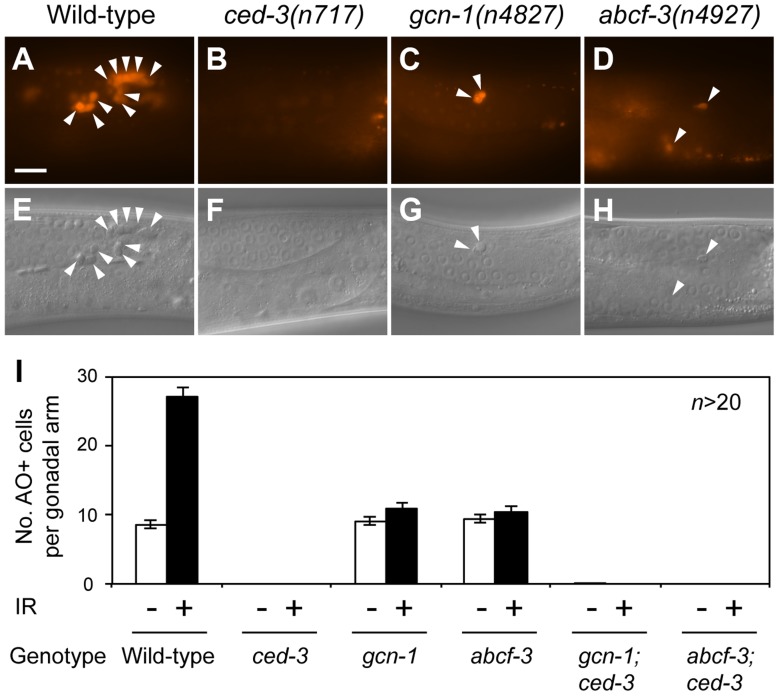
*gcn-1(n4827)* and *abcf-3(n4927)* cause a defect in radiation-induced germline cell death. (A–D) Acridine orange (AO) staining of apoptotic germ cells in the posterior gonads of animals of the indicated genotypes. Arrowheads, AO-positive apoptotic germ cells. Scale bar, 10 µm. (E–H) Nomarski DIC images corresponding to A–D. Arrowheads, refractile apoptotic cells. (I) Numbers of AO-positive apoptotic germ cells in the posterior gonads of animals of the indicated genotypes. White bars, means and standard errors of the means without ionizing radiation (IR). Black bars, means and standard errors of the means with IR.

We next tested whether *gcn-1* and *abcf-3* are involved in the deaths of germ cells in the gonad of the adult hermaphrodite. More than half of germ cells stochastically undergo programmed cell death under normal conditions during oocyte differentiation [37]. We scored the number of apoptotic germ cells using the vital dye acridine orange (AO), which stains nucleic acids within apoptotic cells in living animals [38]. *gcn-1(n4827)* and *abcf-3(n4927)* mutants had 9.1 and 9.5 apoptotic germ cells per gonadal arm on average, respectively, similar to wild-type animals, which had 8.6 apoptotic germ cells per gonadal arm (Figure 4I). We also scored the number of apoptotic germ cells by direct observation of the gonads of engulfment-defective *ced-1(e1735)* mutants, in which cell corpses accumulate because of a defect in cell-corpse engulfment, facilitating a sensitive assay for the deaths of germ cells [37]. *ced-1(e1735)* mutants had an average of 14.4 cell corpses per gonadal arm (Figure S5). *ced-1(e1735)* double mutants with *gcn-1(n4827)* or *abcf-3(n4927)* had nearly identical numbers of cell corpses per gonadal arm, 13.9 and 14.0, respectively (Figure S5). These results indicate that *gcn-1* and *abcf-3* are dispensable for germ-cell death under physiological conditions.

Since many germ cells undergo apoptosis in response to genotoxic stresses such as ionizing radiation [39], we tested whether *gcn-1* and *abcf-3* mediate ionizing radiation damage-induced germ cell death. As assayed with AO, wild-type animals normally contained an average of 8.6 apoptotic germ cells per gonadal arm, while wild-type animals exposed to ionizing radiation contained on average 27.1 apoptotic germ cells (Figure 4A, 4E and 4I). This germ-cell death was completely blocked by a mutation in the caspase gene *ced-3* in wild-type, *gcn-1(n4837)* and *abcf-3(n4927)* animals (Figure 4B, 4F and 4I). Strikingly, ionizing radiation failed to increase the number of apoptotic germ cells in *gcn-1(n4827)* and *abcf-3(n4927)* mutants (10.9 and 10.4 apoptotic germ cells per gonadal arm in *gcn-1* and *abcf-3* mutants, respectively, 24 hours after gamma ray irradiation) (Figure 4C, 4D, 4G, 4H and 4I). These results indicate that *gcn-1* and *abcf-3* are required for ionizing radiation-induced germ cell death but not for the stochastic germ cell death that occurs in physiological conditions.

### *gcn-1* and *abcf-3* gene dosage affects programmed cell death

The *gcn-1(n4827)* and *abcf-3(n4927)* mutations partially blocked both the programmed cell deaths of somatic cells (Table 1) and the deaths of germ cells in response to ionizing radiation (Figure 4). Both somatic and ionizing radiation-induced germ cell deaths involve the canonical cell-death execution pathway consisting of the BH3-only gene *egl-1*, the *BCL-2* homolog *ced-9*, the pro-apoptotic *APAF-1* homolog *ced-4*, and the caspase gene *ced-3* [40]. Interestingly, animals doubly heterozygous for *gcn-1* and *ced-3*, *ced-4* or *egl-1* had a defect in M4 sister cell death (*gcn-1/+; ced-3/+* 18%, *gcn-1/+; ced-4/+* 12% or *gcn-1/+; egl-1/+* 17%, respectively) significantly higher than that of singly heterozygous animals (*gcn-1/+* 4%, *ced-3/+* 0%, *ced-4/+* 0% or *egl-1/+* 1%, respectively) (Table S5). These results indicate that the simultaneous reduction by half of the dosage of *gcn-1* and of genes in the canonical cell-death execution pathway causes a significant defect in M4 sister cell death. We observed a similar genetic interaction in animals heterozygous for *abcf-3* and *ced-3*, *ced-4* or *egl-1* (Table S5).

### *gcn-1* and *abcf-3* maternally contribute to programmed cell death

We observed that maternal *gcn-1* and *abcf-3* contribute to zygotic programmed cell death. While *gcn-1(−)* animals generated by *gcn-1(−)* hermaphrodites and *gcn-1(−)* males exhibited a defect in M4 sister cell death (12% of M4 sister survival), *gcn-1(−)* animals produced from *gcn-1/+* hermaphrodites and *gcn-1(−)* males did not (0% of M4 sister survival) (Table S5), indicating that maternal *gcn-1* is sufficient to promote programmed cell death. *gcn-1(−)* animals generated by *gcn-1(−)* hermaphrodites and *gcn-1/+* males exhibited a defect in M4 sister cell death (13% of M4 sister survival). *gcn-1/+* animals generated by *gcn-1(−)* hermaphrodites and *gcn-1(+)* males exhibited a very weak defect in M4 sister cell death (4% of M4 sister survival) compared to 12% of M4 sister survival in *gcn-1(−)* self-progeny of *gcn-1(−)* hermaphrodites. By contrast, *gcn-1/+* animals produced from *gcn-1(+)* hermaphrodites and *gcn-1(−)* males exhibited no defect in M4 sister cell death (0% of M4 sister survival) (Table S5). These results indicate that maternal *gcn-1* is partially required for the M4 sister to undergo programmed cell death. We observed a similar maternal requirement and sufficiency for *abcf-3* (Table S5). We conclude that maternal *gcn-1* and *abcf-3* are sufficient and partially required for the M4 sister to undergo programmed cell death.

### GCN-1 and ABCF-3 act independently of the BCL-2 homolog CED-9 to promote programmed cell death and can act in the cell fated to die

To examine interactions between *gcn-1* and *abcf-3* and the canonical cell-death execution pathway, we performed epistasis analyses between *gcn-1* or *abcf-3* and *ced-9*, which functions downstream of *egl-1* and upstream of *ced-4* and *ced-3* in the cell-death execution pathway [40]. Because the *ced-9(n2812)* null mutation causes ectopic cell deaths and organismic inviability, we used the *ced-3* partial loss-of-function mutation *n2446* to suppress *ced-9(n2812)* lethality [41]. We observed that 50% of *ced-9(n2812)* animals had a surviving M4 sister in the *ced-3(n2446)* mutant background. This increase over the 5% frequency of M4 sister survival in *ced-3(n2446)* mutants is consistent with the proposal that *ced-9* has a cell-killing activity [42]. We observed that *gcn-1 ced-9* and *abcf-3 ced-9* double mutants were more highly penetrant for M4 sister survival (90% and 89%, respectively) than either single mutant in the *ced-3(n2446)* mutant background: *gcn-1* (45%), *abcf-3* (40%) and *ced-9* (50%), respectively (Table 2). These results indicate that *ced-9* is not required for *gcn-1* and *abcf-3* to promote programmed cell death. Thus, *gcn-1* and *abcf-3* function downstream of or in parallel to *ced-9* in the regulation of programmed cell death.

**Table 2:** *gcn-1(n4827)* and *abcf-3(n4927)* enhance the M4 sister-cell death defect of *ced-9(n2812)* mutants.

Genotype^1^	% M4 sister survival
*ced-3(n2446)*	5
*gcn-1(n4827); ced-3(n2446)*	45
*abcf-3(n4927); ced-3(n2446)*	40
*ced-9(n2812); ced-3(n2446)*	50
*gcn-1(n4827) ced-9(n2812); ced-3(n2446)*	90^2^
*abcf-3(n4927) ced-9(n2812); ced-3(n2446)*	89^2^

1 All strains were homozygous for *nIs177[P_ceh-28_::gfp]*.

2 Fisher's exact test compared with *ced-9(n2812)*, P<0.0001.

120 animals were scored for survival of the M4 sister.

**Figure 5:**
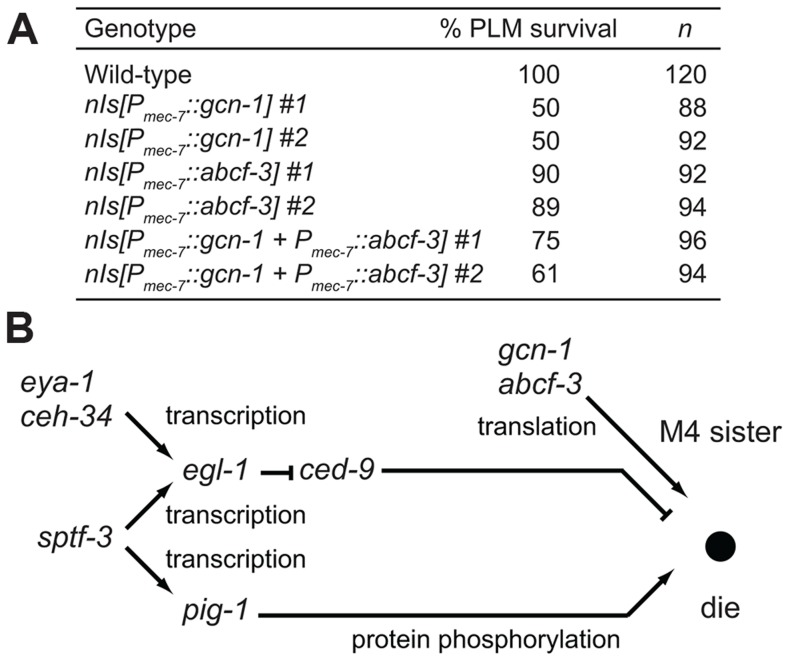
GCN-1 and ABCF-3 act cell-autonomously in a pathway distinct from the canonical cell-death execution pathway. (A) The percentages of PLM survival in animals of the indicated genotypes. All strains had the *P_mec-4_::gfp* transgene that expressed GFP in the touch neurons, including the PLM neurons. (B) A model for the pathways that regulate M4 sister cell-specific programmed cell death. *gcn-1* and *abcf-3* act in a pathway distinct from the canonical *ced-9*-dependent cell-death execution pathway to promote M4 sister cell death. See text for details.

We next tested whether the activity of *gcn-1* and *abcf-3* can act cell-autonomously to promote programmed cell death. Previous studies showed that expression of a *ced-3*, *ced-4* or *egl-1* cDNA under the control of the *mec-7* promoter can act cell-autonomously to cause the deaths of a set of touch neurons, including the PLML and PLMR cells. We expressed *gcn-1* and *abcf-3* cDNAs in the PLM neurons under the control of the *mec-7* promoter. We observed that 100% of the PLM neurons survived in wild-type animals, whereas only 50% or 89% of the PLM neurons survived in animals expressing *gcn-1* or *abcf-3*, respectively, under the control of the *mec-7* promoter (Figure 5A). Expression of both *gcn-1* and *abcf-3* also reduced a survival of the PLM neurons: 61% of the PLM neurons survived. These results indicate that expression of *gcn-1* and *abcf-3* are sufficient to induce cell death and suggest that *gcn-1* and *abcf-3* acts cell-autonomously to promote programmed cell death.

Our genetic screen for mutants defective in M4 sister cell death identified other genes in addition to *gcn-1* and *abcf-3*: *ceh-34*, *eya-1*, *sptf-3* and *pig-1* [15], [17]. We previously showed that the Six family homeodomain protein CEH-34 and the Eyes absent homolog EYA-1 directly drive the transcription of the BH3-only gen*e egl-1* in the M4 sister to promote M4 sister cell-type specific death [15] and that the SP1 family transcription factor SPTF-3 directly drives the transcription of both *egl-1* and the AMPK-related protein kinase gene *pig-1*, which also promotes M4 sister cell death [17]. We determined how *gcn-1* and *abcf-3* interact with these genes by examining double mutants. The partial loss-of-function alleles *ceh-34(n4796)* and *sptf-3(n4850)* and the null allele *pig-1(gm344Δ)* enhanced the M4 sister-cell death defect of *gcn-1(n4827)* and *abcf-3(n4927)* null mutants (Table 3). These results indicate that *ceh-34*, *sptf-3* and *pig-1* function in pathways distinct from that of *gcn-1* and *abcf-3* to promote M4 sister cell death.

**Table 3:** *ceh-34*, *eya-1*, *sptf-3* and *pig-1* function independently of *gcn-1* and *abcf-3*.

Genotype	% M4 sister survival
Wild-type	0
*gnc-1(n4827)*	12
*abcf-3(n4927)*	13
*ceh-34(n4796)*	38^2^
*sptf-3(n4850)*	34
*pig-1(gm344Δ)*	20
*gnc-1(n4827); ceh-34(n4796)* 1	52^3^
*gnc-1(n4827); sptf-3(n4850)*	56^4^
*gnc-1(n4827); pig-1(gm344Δ)*	68^5^
*abcf-3(n4927); ceh-34(n4796)* 1	54^3^
*abcf-3(n4927); sptf-3(n4850)*	58^4^
*abcf-3(n4927); pig-1(gm344Δ)*	63^5^

1 These strains were homozygous for *unc-45(r450)*.

2 Data from ref. 15.

3 Fisher's exact test compared with *ceh-34(n4796)*, *P*<0.05.

4 Fisher's exact test compared with *sptf-3(n4850)*, *P*<0.001.

5 Fisher's exact test compared with *pig-1(gm344Δ)*, *P*<0.0001.

More than 120 animals were scored for survival of the M4 sister.

## Discussion

### The maternally-contributed translational regulators GCN-1 and ABCF-3 act together to promote the death of the M4 sister in a pathway distinct from the CED-9-mediated cell-death execution pathway

We demonstrated that the translational regulators GCN-1 and ABCF-3 are pro-apoptotic factors that maternally contribute to the programmed cell death of the M4 sister in *C. elegans*. GCN-1 and ABCF-3 promote the deaths of all somatic cells tested. Essentially all somatic cell deaths are mediated by an evolutionarily conserved cell-death execution pathway consisting of the BH3-only gene *egl-1*, the *BCL-2* homolog *ced-9*, the *APAF-1* homolog *ced-4* and the caspase gene *ced-3* [40]. How do *gcn-1* and *abcf-3* interact with this pathway to regulate apoptosis? We propose that *gcn-1* and *abcf-3* likely act in a novel pathway distinct from the canonical cell-death execution pathway. First, *gcn-1* and *abcf-*3 promote apoptosis in the absence of *ced-9* activity, indicating that *gcn-1* and *abcf-3* function independently of *ced-9* in the regulation of apoptosis and hence do not regulate either *ced-9* or *egl-1*. Second since *ced-3* and *ced-4* function downstream of *ced-9* in the cell-death execution pathway, *gcn-1* and *abcf-3* could act through these genes to promote cell death. However, our mRNA-seq and Ribo-seq results indicate that *gcn-1* and *abcf-3* do not have major effects on mRNA abundance or the ribosome footprint density of *ced-3* and *ced-4* (Figure S6). Our preferred model is that GCN-1 and ABCF-3 function in a pathway that acts in parallel to the canonical cell-death execution pathway, although we cannot preclude the possibility that GCN-1 and ABCF-3 translationally regulate unidentified factors that act through *ced-3* or *ced-4* without changing the transcriptional and translational levels of the products of these genes. Also, since we used whole animals for our mRNA-seq and Ribo-seq analyses, we would not have detected alterations in CED-3 or CED-4 levels that were specific to a small subset of cells, including the M4 sister.

Our genetic analyses revealed that *gcn-1* and *abcf-3* maternally contribute to the death of the M4 sister, which undergoes programmed cell death during embryogenesis. Maternally-contributed factors might act to ensure the rapid deaths of cells during embryogenesis; perhaps zygotic expression of apoptotic genes would be too slow. Also, the maternal effects of *gcn-1* and *abcf-3* might explain why we discovered new general cell-death genes, despite the fact that many genetic screens have been performed in search of *C. elegans* mutants defective in somatic cell deaths. Most such genetic screens have examined F2 animals after mutagenesis, and would have missed maternally-contributed genes that affect general cell death. Perhaps, additional maternal-effect genes with functions in apoptosis exist in *C. elegans*. Such genes might be efficiently identified by screening in the third generation after mutagenesis.

*gcn-1(n4827)* and *abcf-3(n4927)* single mutations appeared to cause a defect in only M4 sister cell death, and these mutations both affected other cell deaths to differing extents in strains sensitized to weak defects in cell death. For example, the death of the M4 sister was most sensitive and the deaths of the PVQ sisters were least sensitive to the *gcn-1(n4827)* and *abcf-3(n4927)* mutations among the cells we tested (Table 1). We speculate that sensitivity to perturbation of cell-death genes is different among different cell types. This hypothesis is supported by the observations that penetrance of cell-death defects varies among different cell types in partial loss-of-function *ced-3(n2427)* mutants and that the extent of the cell-death defect of *ced-3(n2427)* mutants is well correlated with that of *gcn-1(n4827)* and *abcf-3(n4927)* mutants.

Our genetic and biochemical data strongly suggest that GCN-1 and ABCF-3 physically interact in a complex *in vivo* to promote apoptosis. First, GCN-1 and ABCF-3 interacted in the yeast two-hybrid system. Second, GCN-1 co-immunoprecipitated with ABCF-3 from a total protein extract from *C. elegans*. Third, the absence of either the GCN-1 or ABCF-3 protein decreased the steady-state level of the other protein (ABCF-3 or GCN-1, respectively), indicating that an interaction between GCN-1 and ABCF-3 is likely important for the stability of both proteins. Fourth, *gcn-1(n4827) abcf-3(n4927)* double mutants were not enhanced in the defect in apoptosis compared to each single mutant.

### GCN-1 and ABCF-3 play an essential role in germ-cell death in response to ionizing radiation

Although GCN-1 and ABCF-3 promote the deaths of most somatic cells, in *gcn-1* or *abcf-3* mutants only 12% or 13% of animals are defective in M4 sister cell death, respectively, and the cell-death defect of most other cells was observed only in a partial loss-of-function *ced-3* mutant background, which is sensitized to weak defects in cell death. Furthermore, loss-of-function of *gcn-1* and *abcf-3* did not affect the deaths of germ cells under physiological conditions. By striking contrast, we found that GCN-1 and ABCF-3 play an essential role in germ-cell deaths induced by ionizing radiation. These results suggest that translational control by GCN-1 and ABCF-3 plays a more important role in germ-cell deaths induced by ionizing radiation than in somatic cell deaths. The hypothesis that translational control is particularly important for cell deaths induced by ionizing radiation is supported by a recent report that a mutation in RNA polymerase I (*rpoa-2*), which synthesizes ribosomal RNAs, causes a defect in germ-cell deaths induced by ionizing radiation [43].

Ionizing radiation causes DNA double-strand breaks, which lead to the progressive accumulation of mutations and chromosomal aberrations as damaged cells undergo division, resulting in apoptosis and the demise of genetically damaged cells. In *C. elegans*, ionizing radiation causes massive deaths of the germ cells during the late pachytene stage of oocyte development in adult gonads, resulting in the elimination of the damaged oocytes [39]. Germ-cell deaths induced by ionizing radiation specifically involve activation of the p53 homolog CEP-1 by the DNA damage response pathway and subsequent CEP-1- dependent transcriptional induction of the BH3-only gene *egl-1*, which activates the cell-death execution pathway regulated by CED-9 [44], [45]. How might GCN-1 and ABCF-3 interact with the known DNA-damage response and cell-death execution pathways in the regulation of germline cell deaths induced by ionizing radiation? Our genetic results suggest that GCN-1 and ABCF-3 function independently of CED-9, at least in the regulation of the death of the M4 sister cell. We suggest that as is the case for somatic cell deaths, GCN-1 and ABCF-3 function in a novel pathway independently of CED-9 in regulating the germ-cell deaths induced by ionizing radiation. Alternatively, if GCN-1 and ABCF-3 regulate germ-cell deaths induced by ionizing radiation via a mechanism different from that of somatic cell deaths, it is possible that GCN-1 and ABCF-3 act through *egl-1* and its target *ced-9*, since *egl-1* is involved in somatic programmed cell deaths and germ-cell deaths induced by ionizing radiation but not in the stochastic germ-cell deaths that occur under physiological conditions.

### The functions of GCN-1 and ABCF-3 in the control of translation are conserved between *S. cerevisiae* and *C. elegans*

GCN-1 and ABCF-3 are conserved proteins from yeast to humans. The *C. elegans* GCN-1 protein has 43% and 53% similarities (23% and 32% identities) to the homologs of *S. cerevisiae* and humans, respectively, and the *C. elegans* ABCF-3 proteins has 57% and 69% similarities (40% and 49% identities) to the homologs of *S. cerevisiae* and humans, respectively (Figure 1H). The yeast GCN-1 homolog Gcn1p and ABCF-3 homolog Gcn20p are required to maintain the basal level of the phosphorylation of eukaryotic initiation factor 2α (eIF2α) in the physiological condition and to increase the phosphorylation of eIF2α in response to amino-acid starvation. Gcn1p and Gcn20p activate the serine-threonine protein kinase Gcn2p, which phosphorylates an evolutionarily conserved serine residue of eIF2α. The phosphorylation of eIF2α results in both the inhibition of global translation and the translational activation of the *GCN4* mRNA, which encodes a basic leucine zipper transcription factor. Translation of *GCN4* mRNA is regulated by four short upstream open reading frames (uORFs) in the 5′ UTR with start codons that are out-of-frame with the main coding sequence and which generally reduce translation from the main reading frame [46].

We speculate that the mechanistic roles of *C. elegans* GCN-1 and ABCF-3 in translational control are conserved between yeast and *C. elegans*. First, the amino-acid sequences of GCN-1 and ABCF-3 proteins are conserved between yeast and *C. elegans*, particularly in functionally important domains (Figure 1H). Second, the functions of GCN-1 and ABCF-3 can be substituted with those of *S. cerevisiae GCN1* and *GCN20*, respectively, for the promotion of M4 sister cell death. Third, like their yeast counterparts, *C. elegans* GCN-1 and ABCF-3 are required to maintain the basal level of phosphorylation of eIF2α and physically interact through an EF3-like domain-containing region of GCN-1 and an N-terminal ABCF-3 domain [43]. Fourth, like Gcn20p, the AAA domain ATPase activity of ABCF-3 is not required for its function [32]. Fifth, the *atf-5* gene, the *C. elegans* homolog of *S. cerevisiae GCN4*, has two upstream ORFs that have been shown to inhibit the translation of the *atf-5* mRNA [47].

### A regulatory network involving transcription, translation and protein phosphorylation specifies the death of the M4 sister

We have shown that in addition to *gcn-1* and *abcf-3*, *ceh-34*, *eya-1*, *sptf-3* and *pig-1* function in M4 sister cell death [15], [17]. We previously reported that the Six family homeodomain protein CEH-34 and the Eyes absent homolog EYA-1 physically interact to directly drive expression of the pro-apoptotic BH3-only gene *egl-1* in the M4 sister, leading to the death of the M4 sister (Figure 5B) [15]. We found that the SP1 family transcription factor SPTF-3 directly drives the transcription of the gene *egl-1*, which encodes a BH3-only protein that promotes apoptosis via the CED-3 caspase-mediated canonical cell-death execution pathway [17]. SPTF-3 also directly drives the transcription of the AMPK-related gene *pig-1*, which encodes a protein kinase that functions in a pathway in parallel to the CED-3-mediated canonical cell-death execution pathway. These interactions are shown in Figure 5B.

Our analyses indicate that *gcn-1* and *abcf-3* likely function in a pathway that acts in parallel to those of *pig-1*, *ceh-34* and *sptf-3*. These results are consistent with a model in which GCN-1 and ABCF-3 act independently of CED-9 to promote M4 sister cell death. In short, we propose that the regulatory network for the death of the M4 sister includes at least three different pathways involving translation, transcription and protein phosphorylation (Figure 5B). Each gene in this network (*gcn-1*, *abcf-3*, *sptf-3*, *pig-1*, *egl-1*, *ceh-34* and *eya-1*) has a human counterpart, some of which are implicated in human diseases, including developmental disorders and cancer. We anticipate that further analyses of this regulatory network will both reveal an evolutionarily conserved mechanism of apoptosis shared between *C. elegans* and humans and provide insights concerning how abnormalities in this apoptotic network can lead to human disease.

## Materials and Methods

### *C. elegans* strains

*C. elegans* strains were cultured at 20°C as described [48]. The N2 strain was used as the wild type. The following mutations, integrations and extrachromosomal arrays were used.

LGI: *sptf-3(n4850)*, *eya-1(ok654Δ)*, *nIs177[P_ceh-28_::gfp, lin-15AB(+)]*, *nIs180[P_tdc-1_::gfp, lin-15AB(+)]*, *zdIs5[P_mec-4_::gfp, lin-15AB(+)]*.

LGII: *rol-1(e91)*, *gcn-2(ok871Δ)*, *Y38E10A.8(tm4094Δ)*.

LGIII: *ced-4(n1162)*, *ced-9(n2812)*, *gcn-1(n4827, nc40Δ)*, *abcf-3(n4927, ok2237Δ)*, *unc-45(r450)*, *dpy-18(e364)*, *nIs176[P_ceh-28_::gfp, lin-15AB(+)]*.

LGIV: *ced-3(n717, n2427, n2446)*, *pig-1(gm344Δ)*, *nIs175[P_ceh-28_::gfp, lin-15AB(+)]*.

LGV: *egl-1(n1084 n3082)*, *ceh-34(n4796)*, *oyIs14[sra-6::gfp]*.

LGX: *lin-15(n765)*, *pek-1(ok275Δ)*, *nIs106[P_lin-11_::gfp, lin-15AB(+)]*, *nIs429[P_phat-5_::gfp, lin-15AB(+)]*, *bcIs24[P_tph-1_::gfp, lin-15AB(+)]*.

Unmapped: *nIs460[P_gcn-1_::gfp]*, *nIs488[P_abcf-3_::gfp]*, *nIs645* and *nIs646[P_mec-7_::gcn-1 cDNA, P_mec-7_::abcf-3 cDNA, P_mec-3_::mCherry, rol-6(su1006)]*, *nIs648* and *nIs649[P_mec-7_::gcn-1 cDNA, P_mec-3_::mCherry, rol-6(su1006)]*, *nIs651* and *nIs652[P_mec-7_::abcf-3 cDNA, P_mec-3_::mCherry, rol-6(su1006)]*. Extrachromosomal arrays: *nEx1817* and *nEx1818[P_gcn-1_::gcn-1 cDNA::gcn-1 3′ UTR, P_lin-44_::gfp]*, *nEx1925* and *nEx1926[abcf-3(+), P_lin-44_::gfp]*, *nEx1928* and *nEx1929[abcf-3 K217M, P_lin-44_::gfp]*, *nEx1931* and *nEx1932[abcf-3 K536M, P_lin-44_::gfp]*, *nEx1934* and *nEx1935[abcf-3 K217M K536M, P_lin-44_::gfp]*, *nEx2223* and *nEx2224[P_ceh-34_::eIF2α S49A, P_lin-44_::gfp]*

### Genetic screen and mapping of *gcn-1(n4827)* and *abcf-3(n4927)*

*gcn-1(n4827)* and *abcf-3(n4927)* were isolated from a genetic screen for mutations that cause an extra GFP-positive M4-like cell in animals carrying the *P_ceh-28_::gfp* transgene [15]. Mutagenesis was performed as described [48]. Mutagenized P_0_ animals were allowed to lay eggs, and 144,000 synchronized F_2_ animals were screened with a fluorescence-equipped dissecting microscope. Single nucleotide polymorphisms were used to map *gcn-1(n4827)* and *abcf-3(n4927)* to a 175 kb interval (III: 2,044,521–2,220,200) and a 5.3 Mb interval (III: 5,346,407–10,613,191), respectively [49]. Whole-genome sequencing of *gcn-1(n4827)* mutants was performed using an Illumina/Solexa GAII, according to the instructions of the manufacture. DNA sequencing of the *abcf-3* locus of *abcf-3(n4927)* mutants was performed using an Applied Biosystems 3130×.

### Analyses of defects in programmed cell deaths of specific cells

The programmed cell deaths of specific cells were scored at the indicated stages using the following strains, which express GFP in specific cells. A fluorescence-equipped compound microscope was used to score the programmed cell deaths. M4 sister cell death, *nIs175*, *nIs176* or *nIs177* at the L1 stage. g1A sister cell death, *nIs429* at the L1 stage. PVQ sister cell death, *oyIs14* at the L4 stage [50]. NSM sister cell death, *bcIs24* at the L1 stage [51]. RIM and RIC sister cell death, *nIs180* at the L1 stage. Extra cells in the anterior pharynx were scored using a compound microscope equipped with Nomarski differential interference contrast optics. For physiological germ-cell deaths, germ-cell corpses in gonads of animals 24 hours after the fourth-larval stage were counted by direct observation using Nomarski optics. For ionizing radiation-induced germ-cell deaths, fourth-larval stage animals were exposed to 120 Gy of ionizing radiation, and germ-cell deaths were scored using acridine orange at 24 hours post-irradiation as described [38].

### Plasmid construction

The transgenes *P_ceh-28_::gfp*, *P_tph-1_::gfp* and *sra-6::gfp* are described [15], [50], [51]. The *phat-5* promoter sequence in pGD48 was cloned in pPD122.56 to generate the *P_phat-5_::gfp* transgene [52]. The *P_flp-15_::gfp* transgene contained 2.4 kbp of the 5′ promoter of *flp-15* in pPD122.56. The *P_gcy-37_::gfp* transgene contained 1.1 kbp of 5′ promoter of *gcy-37* in pPD122.56. The *P_tdc-1_::gfp* transgene contained 4.5 kbp of 5′ promoter of *tdc-1* in pPD121.83. The *P_gcn-1_::gcn-1 cDNA::gcn-1 3′UTR* transgene (pTH *gcn-1* cDNA) contained 4.2 kbp of 5′ promoter of *gcn-1*, a full-length *gcn-1* cDNA and 1.0 kbp 3′ of the stop codon of *gcn-1*. The 5′ promoter of *gcn-1*, a full-length *gcn-1* cDNA and the 3′ promoter of *gcn-1* were generated by PCR and fused in pBluescript II using the In-Fusion cloning system (Clontech). The *abcf-3*(+) transgene contained 1.6 kbp of 5′ promoter, the coding region and 0.8 kbp 3′ of the stop codon of *abcf-3* in pBluescript II. The QuickChange II XL Site-Directed Mutagenesis Kit (Stratagene) was used to generate transgenes of *abcf-3 K217M*, *abcf-3 K536M* and *abcf-3 K217M K536M*. *gcn-1* cDNA corresponding to amino acids 1–2634, 1–1760, 1–880, 880–2634, 880–1760 or 1760–2634 of GCN-1 was cloned in pGBKT7. *abcf-3* cDNA corresponding to amino acids 1–712, 1–512, 1–202, 202–712, 512–712 or 202–512 was cloned in pGADT7. The *P_gcn-1_::gfp* transgene contained 4.2 kbp of 5′ promoter of *gcn-1* in pPD122.56. The *P_abcf-3_::gfp* transgene contained 1.6 kbp of 5′ promoter of *abcf-3* in pPD122.56. The *P_ceh-34_::eIF2α* S49A transgene contained 3.8 kbp of 5′ promoter of *ceh-34* and *eIF2α* with a replacement of serine 49 with alanine in pPD49.26. For the *P_mec-7_::gcn-1 cDNA* and *P_mec-7_::abcf-3 cDNA* transgenes, full-length cDNA of *gcn-1* and *abcf-3* were cloned in pPD96.41. Primer sequences used are available from the authors.

### Germline transformation

Germline transformation was performed as described [53]. The *gfp* reporter transgene was injected at 50 µg/ml into *lin-15(n765ts)* animals with 50 µg/ml of pL15EK as a coinjection marker [54]. To rescue the defect in M4 sister cell death, the transgenes pTH *gcn-1* cDNA, *abcf-3(+)*, *abcf-3 K217M*, *abcf-3 K536M* and *abcf-3 K217M K536M* described above were injected at 20 µg/ml into *gcn-1(n4827)* or *abcf-3(n4927)* animals with 50 µg/ml of *P_lin-44_::gfp* as a coinjection marker [55]. To establish transgenic lines carrying the *P_ceh-34_::eIF2α* S49A transgene, the *P_ceh-34_::eIF2α* S49A transgene was injected at 50 µg/ml into *nIs175* animals with 50 µg/ml of *P_lin-44_::gfp* as a coinjection marker. The *P_mec-7_::gcn-1 cDNA* and *P_mec-7_::abcf-3 cDNA* transgenes were injected at 50 µg/ml, respectively, into *zdIs5* animals with 50 µg/ml of pRF4[*rol-6(su1006)*] and 20 µg/ml of the *P_mec-3_::mCherry* transgene as coinjection markers.

### Yeast two-hybrid binding assay

GAL4 fusion constructs were introduced into yeast strain PJ649A as described [56]. Single colonies were streaked and cultured for two days at 30°C on SD plates containing minimal supplements without tryptophan and leucine. Then yeast strains were streaked and cultured for three days at 30°C on SD plates containing minimal supplements without tryptophan, leucine and histidine to test yeast growth.

### Antibody production

Protein fragments corresponding to amino acids 753–857 of GCN-1 and 74–185 of ABCF-3 fused to glutathione S- transferase (GST) were expressed, purified using glutathione Sepharose 4B (Amersham Biosciences) and used to raise rabbit anti-GCN-1 or anti-ABCF-3 antibodies, respectively. Antisera were generated by Pocono Rabbit Farm and Laboratory. Specific antibodies were affinity-purified using identical GCN-1 or ABCF-3 protein fragments fused to maltose-binding protein (MBP) and coupled to Affigel 10 (Bio-Rad).

### Western blots and immunoprecipitation analysis

Protein extracts were prepared from *nIs175*, *gcn-1(n4827); nIs175* and *abcf-3(n4927); nIs175* animals synchronized at the fourth larval stage as described [57]. 10 µg of total protein was loaded onto a 7.5% SDS PAGE gel and then transferred to nitrocellulose membranes. The membranes were probed with anti-GCN-1 or anti-ABCF-3 antibody. Immunocomplexes were detected using HRP-conjugated anti-rabbit IgG secondary antibodies (Invitrogen) followed by chemiluminescence (Western Lightning ECL, PerkinElmer). To determine the level of phosphorylated eIF2α, protein extracts were prepared from *nIs175*, *gcn-1(n4827); nIs175* and *abcf-3(n4927); nIs175* animals synchronized at the fourth larval stage as described [57]. 15 µg of total protein was loaded on a 10% SDS PAGE gel and then transferred to nitrocellulose membranes. The membranes were probed with anti-phospho-eIF2α (Cell Signaling Technology) and anti-eIF2α antibodies [28]. Immunocomplexes were detected as described above.

For immunoprecipitation experiments, protein extracts were prepared from mixed-staged wild-type animals in TNE buffer containing 50 mM Tris-HCl (pH 8.0), 150 mM NaCl, 1 mM EDTA, 1% NP-40, 5 mM β-mercaptoethanol, 10% glycerol. Protein extracts were mixed with either an affinity-purified anti-ABCF-3 antibody or a control IgG at 4°C for 2 hours. Immunocomplexes were recovered using Protein A Sepharose 4 Fast Flow (GE Healthcare Life Sciences) and washed with TNE buffer four times. The recovered immunocomplexes were subjected to western blot analysis using anti-GCN-1 or anti-ABCF-3 antibody.

### Fluorescence *in situ* hybridization

Fluorescence *in situ* hybridization was performed as described [58]. The *gcn-1* and *abcf-3* probes (Biosearch Technologies, Inc) were conjugated to the fluorophore Cy5 using the Amersham Cy5 Mono-reactive Dye pack (GE Healthcare). DNA was visualized using 4′,6-diamidino-2-phenylindole (DAPI). The probe sequences used are shown in Tables S3 and S4. Figures 4D and H are maximum intensity projections of a Z-stack of images processed with the FFT Bandpass Filter operations in the image processing program Fiji. Oligonucleotides used for *gcn-1* and *abcf-3* FISH probe were described in Table S6 and S7).

### mRNA-seq and ribosome profiling (Ribo-seq)

For mRNA-seq [59], total RNA was purified using an RNAeasy Mini kit (Qiagen) from synchronized L4 animals of wild-type animals and *gcn-1* and *abcf-3* mutants. The purified RNA was subjected to oligo (dT) selection, fragmentation and first- and double-strand synthesis with an Illumina Tru-Seq kit according to the manufacturer's instructions. DNA fragments longer than 30 bp were purified using SPRI-TE beads (Beckmann Coulter) according to the manufacturer's instructions. The purified DNA was end-repaired and single A bases were added for adaptor ligations. The adaptor-ligated DNA was then subjected to double SPRI-TE purification to select for 200 bp fragments. These fragments were enriched and barcoded by PCR for multiplexing. A final SPRI-TE purification was performed to purify the barcoded RNA-Seq libraries for Illumina DNA sequencing using HiSeq 2000. RNA-seq data were aligned against the *C. elegans* reference genome (ce10) using the Burrows-Wheeler Aligner (BWA) and Tophat.

Ribosome profiling was performed as described [60] with modifications. Synchronized L4 wild type animals and *gcn-1* and *abcf-3* mutants were collected and washed with M9 buffer three times. Animals were homogenized using a dounce homogenizer in lysis buffer containing 20 mM Tris (pH 7.5), 150 mM NaCl, 5 mM MgSO_4_, 1 mM DTT, 100 µg/ml cycloheximide, 1% Triton X-100 and 25 U/ml Turbo DNase (Invitrogen) and centrifuged at 20,000 g for 20 min at 4°C. The absorbance of the extract was measured at 260 nm. 40 absorbance units of extract were incubated with 300 units of RNase I at 25°C for an hour, and then 200 units of SUPERase In RNase Inhibitor (Invitrogen) were added. Digested extracts were loaded on 10–50% linear sucrose gradients containing 20 mM Tris (pH 7.5), 150 mM NaCl, 5 mM MgSO_4_, 1 mM DTT and 100 µg/ml cycloheximide and centrifuged for three hours at 35, 000 rpm at 4°C using a SW-40 rotor to isolate a monosome fraction. RNA from the monosome fraction was purified by phenol-chloroform extraction followed by miRNeasy Mini Kit (Qiagen) and separated using a 15% TBE-Urea gel (BioRad) to isolate ribosome-protected fragments (RPFs). The RPFs were eluted from gels by incubating in RNA elution buffer containing 300 mM sodium acetate (pH 5.5), 1 mM EDTA and 0.25% SDS. RPFs were 3′ dephosphorylated with T4 polynucleotide kinase (New England Labs) and ligated to Universal miRNA Cloning Linker (New England Labs) using T4 RNA ligase 2, truncated (New England Labs) according to the manufacturer's instructions. RPFs ligated with the linker were separated from an unligated linker using a 15% TBE-Urea gel (BioRad) and eluted from gels using RNA extraction buffer followed by phenol-chloroform extraction. RFPs were reverse-transcribed by Superscript III (Invitrogen) with a reverse transcription primer according to the manufacturer's instructions. The products of reverse transcripts (RT) were purified using a 15% TBE-Urea gel (BioRad) and eluted from a gel by incubating in DNA elution buffer containing 300 mM NaCl, 10 mM Tris (pH 8.0) and 1 mM EDTA followed by phenol-chloroform extraction. The RT products were circularized by CircLigase (Epicentre) according to the manufacturer's instructions. About a quarter of the RT products were used in PCR reactions containing 1× Phusion HF buffer, 0.2 mM dNTP, 0.5 µm forward library primer, 0.5 µm reverse indexed primer and 0.02 units/µl Phusion polymerase (New England Labs), and PCR was performed with a 30 second initial denaturation at 98°C, followed by 6, 8, 10, 12 and 14 cycles of 98°C for 10 second, 65°C for 10 second and 72°C for 5 second. PCR products were separated using a 8% TBE gel (BioRad) and eluted from gels by incubating in DNA elution buffer followed by phenol-chloroform extraction. PCR products were suspended in 20 µl of 10 mM Tris (pH 8.0) and sequenced by HiSeq 2000. The adaptor sequences (CTGTAGGCACCATC) from 3′ end of the ribosome footprint reads were removed, then trimmed reads were mapped using BWA to distinguish the reads from ribosomal RNAs. About 60% of the reads were filtered out, and the remaining reads (non-ribosomal) were aligned to the *C. elegans* reference genome (ce10) using BWA and Tophat. Because translational initiation is thought to be blocked rapidly by the stress animals encounter during harvesting, many ribosomes are stalled at the beginning of each transcript in the presence of cycloheximide, which prevents translation elongation [34]. Hence, high frequencies of reads at the beginning of each transcript might not correspond to high rates of translation. For this reason, the reads that mapped to the first 25 nucleotides of each transcript were not counted in evaluating gene expression in the Ribo-seq analyses.

## Supporting Information

Figure S1 Genomic positions of *gcn-1* and *abcf-3*. (A) *gcn-1(n4827)* mapped to a 175 kb interval between nucleotides 2,044,521 and 2,220,000 on chromosome III. Arrowheads, mutations found by whole-genome sequencing within the interval. Black arrowhead, mutation located in non-coding region. Red arrowhead, *n4827*. Black bar, *gcn-1* gene. Nucleotide numbers are genomic positions based on WormBase WS53. (B) *abcf-3(n4927)* mapped to a 5.3 Mb interval between nucleotides 5,346,407 and 10,613,191 of chromosome III. The positions of the *abcf-3* and *ced-9* loci are indicated.
(EPS)

Figure S2 Alignment of the *C. elegans* GCN-1 protein sequence with those of yeast Gcn1p and human GCN1. The CLUSTAL W algorithm was used to align the amino acid sequences. Identical amino acids and similar amino acids are indicated with black boxes and gray boxes, respectively. Red arrowhead, position of the amino acid altered by *n4827*. Orange underline, EF3-like domain.
(EPS)

Figure S3 Alignment of *C. elegans* ABCF-3 protein sequence with those of yeast Gcn20p and human ABCF3. The CLUSTAL W algorithm was used to align the amino acids. Identical amino acids and similar amino acids are indicated with black boxes and gray boxes, respectively. Red arrowhead, position of the amino acid altered by *n4927*. Green underline, extent of the AAA domains.
(EPS)

Figure S4 *gcn-1* and *abcf-3* affect the expression of similar sets of genes. (A) Biplots showing log_2_-fold changes of rpkm (reads per kilobase per million) in mRNA-seq (*x*-axis) and Ribo-seq (*y* axis) between wild-type animals and *gcn-1* mutants. (B) Biplots showing log_2_-fold changes of rpkm in mRNA-seq (*x*-axis) and Ribo-seq (*y* axis) between wild-type animals and *abcf-3* mutants. (C and D) Heatmap displaying genes with changes of rpkm in both *gcn-1* and *abcf-3* mutants compared to wild-type animals (*p*<10^−5^) in mRNA-seq and Ribo-seq. Log_2_-fold changes in expression of these genes were hierarchically clustered using a Euclidean distance metric. (E and F) Venn diagram showing overlap of differentially-regulated genes (>2 fold, *p*<0.1) in *gcn-1* and *abcf-3* mutants compared to wild-type animals based on mRNA-seq or Ribo-seq analyses.
(EPS)

Figure S5 *gcn-1* and *abcf-3* do not affect germ-cell death in physiological conditions. Number of apoptotic cell corpses in the gonads of animals of the indicated genotypes at 24 hours after the fourth-larval stage (L4) as visualized using Nomarski optics. Black bars, means. Errors, standard deviations.
(EPS)

Figure S6 *gcn-1* and *abcf-3* do not have a major effect on the translational efficiency of *ced-3* and *ced-4*. Normalized reads (rpkm) of *ced-3*, *ced-4* and *npr-4* in mRNA-seq and Ribo-seq analyses are shown for wild-type animals and *gcn-1* and *abcf-3* mutants.
(EPS)

Table S1 mRNA-seq and ribosome profiling analyses of wild-type animals and *gcn-1* and *abcf-3* mutants.
(XLSX)

Table S2 A list of genes for which transcription changes more than two-fold in *gcn-1* and *abcf-3* mutants compared to the wild type (*p*<0.1).
(XLSX)

Table S3 A list of genes for which translation changes more than two-fold in *gcn-1* and *abcf-3* mutants compared to the wild type (*p*<0.1).
(XLSX)

Table S4 Presumptive eIF2α kinases GCN-2, PEK-1 and Y38E10A.8 do not affect the death of the M4 sister.
(DOCX)

Table S5 In controlling M4 sister cell death, *gcn-1* and *abcf-3* maternally contribute and genetically interact with genes in the cell-death execution pathway.
(DOCX)

Table S6 Oligonucleotides used for *gcn-1* FISH probe.
(DOCX)

Table S7 Oligonucleotides used for *abcf-3* FISH probe.
(DOCX)

## References

[1] Fuchs Y, Steller H (2011) Programmed cell death in animal development and disease. Cell 147: 742–758 10.1016/j.cell.2011.10.033 22078876 PMC4511103

[2] Conradt B (2009) Genetic control of programmed cell death during animal development. Annu Rev Genet 43: 493–523 10.1146/annurev.genet.42.110807.091533 19886811 PMC2806233

[3] Hyman BT, Yuan J (2012) Apoptotic and non-apoptotic roles of caspases in neuronal physiology and pathophysiology. Nat Rev Neurosci 13: 395–406 10.1038/nrn3228 22595785

[4] Hipfner DR, Cohen SM (2004) Connecting proliferation and apoptosis in development and disease. Nat Rev Mol Cell Biol 5: 805–815 10.1038/nrm1491 15459661

[5] Miyashita T, Reed JC (1995) Tumor suppressor p53 is a direct transcriptional activator of the human *bax* gene. Cell 80: 293–299. 7834749 10.1016/0092-8674(95)90412-3

[6] Oda E, Ohki R, Murasawa H, Nemoto J, Shibue T, et al (2000) Noxa, a BH3-only member of the Bcl-2 family and candidate mediator of p53-induced apoptosis. Science 288: 1053–1058. 10807576 10.1126/science.288.5468.1053

[7] Sax JK, Fei P, Murphy ME, Bernhard E, Korsmeyer SJ, et al (2002) *BID* regulation by p53 contributes to chemosensitivity. Nat Cell Biol 4: 842–849 10.1038/ncb866 12402042

[8] Moroni MC, Hickman ES, Lazzerini Denchi E, Caprara G, Colli E, et al (2001) *Apaf-1* is a transcriptional target for E2F and p53. Nat Cell Biol 3: 552–558 10.1038/35078527 11389439

[9] Wu GS, Burns TF, McDonald ER 3rd, Jiang W, Meng R, et al (1997) *KILLER/DR5* is a DNA damage-inducible p53-regulated death receptor gene. Nat Genet 17: 141–143 10.1038/ng1097-141 9326928

[10] Nakano K, Vousden KH (2001) *PUMA*, a novel proapoptotic gene, is induced by p53. Mol Cell 7: 683–694. 11463392 10.1016/s1097-2765(01)00214-3

[11] Schuler M, Green DR (2005) Transcription, apoptosis and p53: catch-22. Trends Genet 21: 182–187 10.1016/j.tig.2005.01.001 15734577

[12] Nehme R, Conradt B (2008) *egl-1*: a key activator of apoptotic cell death in *C. elegans* . Oncogene 27 Suppl 1: S30–40 10.1038/onc.2009.41 19641505

[13] Winn J, Carter M, Avery L, Cameron S (2011) Hox and a newly identified E2F co-repress cell death in *Caenorhabditis elegans* . Genetics 188: 897–905 10.1534/genetics.111.128421 21596899 PMC3176086

[14] Potts MB, Wang DP, Cameron S (2009) Trithorax, Hox, and TALE-class homeodomain proteins ensure cell survival through repression of the BH3-only gene *egl-1* . Dev Biol 329: 374–385 10.1016/j.ydbio.2009.02.022 19254707

[15] Hirose T, Galvin BD, Horvitz HR (2010) Six and Eya promote apoptosis through direct transcriptional activation of the proapoptotic BH3-only gene *egl-1* in *Caenorhabditis elegans* . Proc Natl Acad Sci USA 107: 15479–15484 10.1073/pnas.1010023107 20713707 PMC2932601

[16] Maurer CW, Chiorazzi M, Shaham S (2007) Timing of the onset of a developmental cell death is controlled by transcriptional induction of the *C. elegans ced-3* caspase-encoding gene. Development 134: 1357–1368 10.1242/dev.02818 17329362

[17] Hirose T, Horvitz HR (2013) An Sp1 transcription factor coordinates caspase-dependent and -independent apoptotic pathways. Nature 500: 354–358 10.1038/nature12329 23851392 PMC3748152

[18] Holcik M, Sonenberg N (2005) Translational control in stress and apoptosis. Nat Rev Mol Cell Biol 6: 318–327 10.1038/nrm1618 15803138

[19] Nevins TA, Harder ZM, Korneluk RG, Holcík M (2003) Distinct regulation of internal ribosome entry site-mediated translation following cellular stress is mediated by apoptotic fragments of eIF4G translation initiation factor family members eIF4GI and p97/DAP5/NAT1. J Biol Chem 278: 3572–3579 10.1074/jbc.M206781200 12458215

[20] Holcik M, Lefebvre C, Yeh C, Chow T, Korneluk RG (1999) A new internal-ribosome-entry-site motif potentiates XIAP-mediated cytoprotection. Nat Cell Biol 1: 190–192 10.1038/11109 10559907

[21] Schumacher B, Hanazawa M, Lee M-H, Nayak S, Volkmann K, et al (2005) Translational repression of *C. elegans* p53 by GLD-1 regulates DNA damage-induced apoptosis. Cell 120: 357–368 10.1016/j.cell.2004.12.009 15707894

[22] Contreras V, Friday AJ, Morrison JK, Hao E, Keiper BD (2011) Cap-independent translation promotes *C. elegans* germ cell apoptosis through Apaf-1/CED-4 in a caspase-dependent mechanism. PLoS ONE 6: e24444 10.1371/journal.pone.0024444 21909434 PMC3164730

[23] Contreras V, Richardson MA, Hao E, Keiper BD (2008) Depletion of the cap-associated isoform of translation factor eIF4G induces germline apoptosis in *C. elegans* . Cell Death Differ 15: 1232–1242 10.1038/cdd.2008.46 18451872

[24] Huang C-Y, Chen J-Y, Wu S-C, Tan C-H, Tzeng R-Y, et al (2012) *C. elegans* EIF-3.K promotes programmed cell death through CED-3 caspase. PLoS ONE 7: e36584 10.1371/journal.pone.0036584 22590572 PMC3348885

[25] Avery L, Horvitz HR (1987) A cell that dies during wild-type *C. elegans* development can function as a neuron in a *ced-3* mutant. Cell 51: 1071–1078. 3690660 10.1016/0092-8674(87)90593-9 PMC3773210

[26] Sulston JE, Schierenberg E, White JG, Thomson JN (1983) The embryonic cell lineage of the nematode *Caenorhabditis elegans* . Dev Biol 100: 64–119. 6684600 10.1016/0012-1606(83)90201-4

[27] Genome sequence of the nematode *C. elegans*: a platform for investigating biology. Science 282: 2012–2018. 10.1126/science.282.5396.2012 9851916

[28] Nukazuka A, Fujisawa H, Inada T, Oda Y, Takagi S (2008) Semaphorin controls epidermal morphogenesis by stimulating mRNA translation via eIF2alpha in *Caenorhabditis elegans* . Genes Dev 22: 1025–1036 10.1101/gad.1644008 18413715 PMC2335324

[29] Vazquez de Aldana CR, Marton MJ, Hinnebusch AG (1995) GCN20, a novel ATP binding cassette protein, and GCN1 reside in a complex that mediates activation of the eIF-2 alpha kinase GCN2 in amino acid-starved cells. EMBO J 14: 3184–3199. 7621831 10.1002/j.1460-2075.1995.tb07321.x PMC394380

[30] Marton MJ, Crouch D, Hinnebusch AG (1993) GCN1, a translational activator of *GCN4* in *Saccharomyces cerevisiae*, is required for phosphorylation of eukaryotic translation initiation factor 2 by protein kinase GCN2. Mol Cell Biol 13: 3541–3556. 8497269 10.1128/mcb.13.6.3541-3556.1993 PMC359824

[31] Hanson PI, Whiteheart SW (2005) AAA+ proteins: have engine, will work. Nat Rev Mol Cell Biol 6: 519–529 10.1038/nrm1684 16072036

[32] Marton MJ, Vazquez de Aldana CR, Qiu H, Chakraburtty K, Hinnebusch AG (1997) Evidence that GCN1 and GCN20, translational regulators of *GCN4*, function on elongating ribosomes in activation of eIF2alpha kinase GCN2. Mol Cell Biol 17: 4474–4489. 9234705 10.1128/mcb.17.8.4474 PMC232301

[33] Davison EM, Saffer AM, Huang LS, DeModena J, Sternberg PW, et al (2011) The LIN-15A and LIN-56 transcriptional regulators interact to negatively regulate EGF/Ras signaling in *Caenorhabditis elegans* vulval cell-fate determination. Genetics 187: 803–815 10.1534/genetics.110.124487 21196525 PMC3063674

[34] Ingolia NT, Ghaemmaghami S, Newman JRS, Weissman JS (2009) Genome-wide analysis *in vivo* of translation with nucleotide resolution using ribosome profiling. Science 324: 218–223 10.1126/science.1168978 19213877 PMC2746483

[35] Raj A, Rifkin SA, Andersen E, Van Oudenaarden A (2010) Variability in gene expression underlies incomplete penetrance. Nature 463: 913–918 10.1038/nature08781 20164922 PMC2836165

[36] Reddien PW, Cameron S, Horvitz HR (2001) Phagocytosis promotes programmed cell death in *C. elegans* . Nature 412: 198–202 10.1038/35084096 11449278

[37] Gumienny TL, Lambie E, Hartwieg E, Horvitz HR, Hengartner MO (1999) Genetic control of programmed cell death in the *Caenorhabditis elegans* hermaphrodite germline. Development 126: 1011–1022. 9927601 10.1242/dev.126.5.1011

[38] Lettre G, Kritikou EA, Jaeggi M, Calixto A, Fraser AG, et al (2004) Genome-wide RNAi identifies p53-dependent and -independent regulators of germ cell apoptosis in *C. elegans* . Cell Death Differ 11: 1198–1203 10.1038/sj.cdd.4401488 15272318

[39] Gartner A, Milstein S, Ahmed S, Hodgkin J, Hengartner MO (2000) A conserved checkpoint pathway mediates DNA damage–induced apoptosis and cell cycle arrest in *C. elegans* . Mol Cell 5: 435–443. 10882129 10.1016/s1097-2765(00)80438-4

[40] Metzstein MM, Stanfield GM, Horvitz HR (1998) Genetics of programmed cell death in *C. elegans*: past, present and future. Trends Genet 14: 410–416. 9820030 10.1016/s0168-9525(98)01573-x

[41] Hengartner MO, Ellis RE, Horvitz HR (1992) *Caenorhabditis elegans* gene *ced-9* protects cells from programmed cell death. Nature 356: 494–499 10.1038/356494a0 1560823

[42] Hengartner MO, Horvitz HR (1994) Activation of *C. elegans* cell death protein CED-9 by an amino-acid substitution in a domain conserved in Bcl-2. Nature 369: 318–320 10.1038/369318a0 7910376

[43] Eberhard R, Stergiou L, Hofmann ER, Hofmann J, Haenni S, et al (2013) Ribosome synthesis and MAPK activity modulate ionizing radiation-induced germ cell apoptosis in *Caenorhabditis elegans* . PLoS Genet 9: e1003943 10.1371/journal.pgen.1003943 24278030 PMC3836707

[44] Schumacher B, Hofmann K, Boulton S, Gartner A (2001) The *C. elegans* homolog of the p53 tumor suppressor is required for DNA damage-induced apoptosis. Curr Biol 11: 1722–1727. 11696333 10.1016/s0960-9822(01)00534-6

[45] Hofmann ER, Milstein S, Boulton SJ, Ye M, Hofmann JJ, et al (2002) *Caenorhabditis elegans* HUS-1 is a DNA damage checkpoint protein required for genome stability and EGL-1-mediated apoptosis. Curr Biol 12: 1908–1918. 12445383 10.1016/s0960-9822(02)01262-9

[46] Miller PF, Hinnebusch AG (1990) cis-acting sequences involved in the translational control of *GCN4* expression. Biochim Biophys Acta 1050: 151–154. 2207139 10.1016/0167-4781(90)90157-w

[47] Rousakis A, Vlassis A, Vlanti A, Patera S, Thireos G, et al (2013) The general control nonderepressible-2 kinase mediates stress response and longevity induced by target of rapamycin inactivation in Caenorhabditis elegans. Aging Cell 12: 742–51 10.1111/acel.12101 23692540 PMC4225475

[48] Brenner S (1974) The genetics of *Caenorhabditis elegans* . Genetics 77: 71–94. 4366476 10.1093/genetics/77.1.71 PMC1213120

[49] Wicks SR, Yeh RT, Gish WR, Waterston RH, Plasterk RH (2001) Rapid gene mapping in *Caenorhabditis elegans* using a high density polymorphism map. Nat Genet 28: 160–164 10.1038/88878 11381264

[50] Troemel ER, Chou JH, Dwyer ND, Colbert HA, Bargmann CI (1995) Divergent seven transmembrane receptors are candidate chemosensory receptors in *C. elegans* . Cell 83: 207–218. 7585938 10.1016/0092-8674(95)90162-0

[51] Thellmann M, Hatzold J, Conradt B (2003) The Snail-like CES-1 protein of *C. elegans* can block the expression of the BH3-only cell-death activator gene *egl-1* by antagonizing the function of bHLH proteins. Development 130: 4057–4071. 12874127 10.1242/dev.00597

[52] Smit RB, Schnabel R, Gaudet J (2008) The HLH-6 transcription factor regulates *C. elegans* pharyngeal gland development and function. PLoS Genet 4: e1000222 10.1371/journal.pgen.1000222 18927627 PMC2563036

[53] Mello CC, Kramer JM, Stinchcomb D, Ambros V (1991) Efficient gene transfer in *C. elegans*: extrachromosomal maintenance and integration of transforming sequences. EMBO J 10: 3959–3970. 1935914 10.1002/j.1460-2075.1991.tb04966.x PMC453137

[54] Clark SG, Lu X, Horvitz HR (1994) The *Caenorhabditis elegans* locus *lin-15*, a negative regulator of a tyrosine kinase signaling pathway, encodes two different proteins. Genetics 137: 987–997. 7982579 10.1093/genetics/137.4.987 PMC1206075

[55] Herman MA, Vassilieva LL, Horvitz HR, Shaw JE, Herman RK (1995) The *C. elegans* gene *lin-44*, which controls the polarity of certain asymmetric cell divisions, encodes a Wnt protein and acts cell nonautonomously. Cell 83: 101–110. 7553861 10.1016/0092-8674(95)90238-4

[56] Knop M, Siegers K, Pereira G, Zachariae W, Winsor B, et al (1999) Epitope tagging of yeast genes using a PCR-based strategy: more tags and improved practical routines. Yeast 15: 963–972 doi:;10.1002/(SICI)1097-0061(199907)15:10B<963::AID-YEA399>3.0.CO;2-W 10407276 10.1002/(SICI)1097-0061(199907)15:10B<963::AID-YEA399>3.0.CO;2-W

[57] Youngman MJ, Rogers ZN, Kim DH (2011) A decline in p38 MAPK signaling underlies immunosenescence in *Caenorhabditis elegans* . PLoS Genet 7: e1002082 10.1371/journal.pgen.1002082 21625567 PMC3098197

[58] Raj A, Van den Bogaard P, Rifkin SA, Van Oudenaarden A, Tyagi S (2008) Imaging individual mRNA molecules using multiple singly labeled probes. Nat Methods 5: 877–879 10.1038/nmeth.1253 18806792 PMC3126653

[59] Subramanian V, Mazumder A, Surface LE, Butty VL, Fields PA, et al (2013) H2A.Z acidic patch couples chromatin dynamics to regulation of gene expression programs during ESC differentiation. PLoS Genet 9: e1003725 10.1371/journal.pgen.1003725 23990805 PMC3749939

[60] Ingolia NT, Brar GA, Rouskin S, McGeachy AM, Weissman JS (2012) The ribosome profiling strategy for monitoring translation *in vivo* by deep sequencing of ribosome-protected mRNA fragments. Nat Protoc 7: 1534–1550 10.1038/nprot.2012.086 22836135 PMC3535016

