# Supplementary material for: The Translational Regulators GCN-1 and ABCF-3 Act Together to Promote Apoptosis in C. elegans
Source: PLoS Genet. 2014 Aug 7;10(8):e1004512. doi: 10.1371/journal.pgen.1004512 (PMC4125083; doi:10.1371/journal.pgen.1004512)
Supplement: Table S4 — Presumptive eIF2α kinases GCN-2, PEK-1 and Y38E10A.8 do not affect the death of the M4 sister. (DOCX) [file pgen.1004512.s010.docx]

**Table S4. Presumptive eIF2α kinases GCN-2, PEK-1 and Y38E10A.8 do not affect the death of the M4 sister.**

| Genotype | % M4 sister survival | *n* |
| --- | --- | --- |
| Wild-type | 0 | 120 |
| *gcn-2(ok871Δ)* | 0 | 60 |
| *pek-1(ok275Δ)* | 0 | 60 |
| *Y38E10A.8(tm4094Δ)* | 0 | 60 |
| *Y38E10A.8(tm4094Δ) gcn-2(ok871Δ); pek-1(ok275Δ)** | 0 | 60 |

All strains were homozygous for *nIs175[P_ceh-28_::gfp].*

*This strain was homozygous for *rol-1(e91).*
