# Supplementary material for: The Translational Regulators GCN-1 and ABCF-3 Act Together to Promote Apoptosis in C. elegans
Source: PLoS Genet. 2014 Aug 7;10(8):e1004512. doi: 10.1371/journal.pgen.1004512 (PMC4125083; doi:10.1371/journal.pgen.1004512)
Supplement: Table S5 — In controlling M4 sister cell death, gcn-1 and abcf-3 maternally contribute and genetically interact with genes in the cell-death execution pathway. (DOCX) [file pgen.1004512.s011.docx]

**Table S5. In controlling M4 sister cell death, *gcn-1* and *abcf-3* maternally contribute and genetically interact with genes in the cell-death execution pathway.**

| Maternal genotype | Paternal genotype | Zygotic genotype | % M4 sister survival | *n* |
| --- | --- | --- | --- | --- |
| + | + | +/+ | 0 | 120 |
| *gcn-1* | *gcn-1* | *gcn-1/gcn-1* | 12 | 120 |
| *gcn-1/+* | *gcn-1* | *gcn-1/gcn-1* | 0 | 120 |
| *gcn-1* | *gcn-1/+* | *gcn-1/gcn-1* | 13 | 120 |
| *gcn-1* | + | *gcn-1/+* | 4 | 120 |
| + | *gcn-1* | *+/gcn-1* | 0 | 120 |
| + | *ced-3* | *+/ced-3* | 0 | 100 |
| + | *ced-4* | *+/ced-4* | 0 | 100 |
| + | *egl-1* | *+/egl-1* | 1 | 100 |
| *gcn-1* | *ced-3* | *gcn-1/+; ced-3/+* | 18^1^ | 100 |
| *gcn-1* | *ced-4* | *gcn-1 ced-4/+ +* | 12^1^ | 100 |
| *gcn-1* | *egl-1* | *gcn-1/+; egl-1/+* | 17^1^ | 100 |
|  |  |  |  |  |
| *abcf-3* | *abcf-3* | *abcf-3/abcf-3* | 13 | 120 |
| *abcf-3/+* | *abcf-3* | *abcf-3/abcf-3* | 0 | 120 |
| *abcf-3* | *abcf-3/+* | *abcf-3/abcf-3* | 11 | 120 |
| *abcf-3* | + | *abcf-3/+* | 5 | 100 |
| + | *abcf-3* | *+/abcf-3* | 0 | 100 |
| *abcf-3* | *ced-3* | *abcf-3/+; ced-3/+* | 19^2^ | 100 |
| *abcf-3* | *ced-4* | *abcf-3 ced-4/+ +* | 19^2^ | 100 |
| *abcf-3* | *egl-1* | *abcf-3/+; egl-1/+* | 16^2^ | 100 |

Alleles used were the following; *gcn-1(n4827), ced-3(n717), ced-4(n1162)* and *egl-1(n1084 n3082).*

All strains carried *nIs175[P_ceh-28_::gfp], nIs176[P_ceh-28_::gfp]* or *nIs177[P_ceh-28_::gfp].*

F1 male animals of the indicated zygotic genotype were scored for survival of the M4 sister.

^1^Fisher's exact test compared with *gcn-1/+* animals produced from *gcn-1* hermaphrodites and wild-type males*, P*<0.05.

^2^Fisher's exact test compared with *abcf-3/+* animals produced from *abcf-3* hermaphrodites and wild-type males*, P*<0.05.
