# Supplementary material for: The Translational Regulators GCN-1 and ABCF-3 Act Together to Promote Apoptosis in C. elegans
Source: PLoS Genet. 2014 Aug 7;10(8):e1004512. doi: 10.1371/journal.pgen.1004512 (PMC4125083; doi:10.1371/journal.pgen.1004512)
Supplement: Table S6 — Oligonucleotides used for gcn-1 FISH probe. (DOCX) [file pgen.1004512.s012.docx]

| Table S6: Oligonucleotides used for *gcn-1* FISH probe | |
| --- | --- |
| ctcctgaacatgttcgcttt | atccggcgatttttggagaa |
| ttcctttaatgcgtcctcct | ttggcagcaaattcgtctca |
| tcgaaactgccgacgaaaaa | cggagcagtgcttttttcat |
| attgagccggaatttccgtt | ttccgaaaatcgcgacttca |
| aatgctcattttgacgagcc | atcctgagcacaaaagtcga |
| attttggctgtccatagagc | gcgaggaaatcgtagatttg |
| caattttgagacggcctgaa | attccagcttcacgaatctc |
| tttgagtgtggagatgggat | ttttcggcttaaagccacca |
| tggtgattgtttggagcagt | gcgtgttgagaatcttctga |
| tttgggatgattggaagagc | tgcagccgaaaattgatcga |
| tgtgagagactatgggattc | agcacttaatttccgaggct |
| gccaaaagccatttcatcgt | attgctccaacaccttccat |
| gcaggaatttccatccaaac | atatcctgagcttccacaac |
| ctccaaagatttccacagca | gccgtctttgaaatcacatc |
| acatccaataggcgattgct | gtgagattctttatccgcct |
| acgccaagctttatcactag | cagcattccattgagcttca |
| gagccagcttctttgtgaaa | acagtcgaattgcccattct |
| tcgggatttttcgcgaaaag | gtcagtgaaattgccggttt |
| ccgatttttgcggtgaaaac | tttcgaagcctctttgaacg |
| cgatttctcgggattttcct | tccgatatatttgacggctg |
| aatttcaggagttttcgcgg | aaatgtccgccaaaactcga |
| gagaagctgggattttgtga | tgacgtcatcgatcttcgtt |
| tgacgaaaaattgcggtggt | agcaaagttcgccttttggt |
| accgcgaatttcgagattct | cctttttggtggttcgaaga |
