# Supplementary material for: The Translational Regulators GCN-1 and ABCF-3 Act Together to Promote Apoptosis in C. elegans
Source: PLoS Genet. 2014 Aug 7;10(8):e1004512. doi: 10.1371/journal.pgen.1004512 (PMC4125083; doi:10.1371/journal.pgen.1004512)
Supplement: Table S7 — Oligonucleotides used for abcf-3 FISH probe. (DOCX) [file pgen.1004512.s013.docx]

| Table S7: Oligonucleotides used for *abcf-3* FISH probe | |
| --- | --- |
| gccaggaattgcttcaacat | gtctcttcgtatctgacatc |
| ttggaagtgtgggaaaagtg | gcgtcttttccctatcaaga |
| gtgatctcatcggaattctc | ggcgtcattgatattgtcct |
| cggcatccataacttcatct | gctcgtcattccatttggtt |
| gaagcttgaagatgttcacc | gagagcttccatttcgatgt |
| caacctcgcaaagattttcc | ataaagaagggaagaagccc |
| ccgtgaagtagtttcagaag | aactcctttgttggacgctt |
| gatagcctttggaaggttgt | atctggcttcacgaatagag |
| tcgtagttgcttcctgttgt | ttagtcggttcgtcaagcaa |
| tgtcaacagtttgctcgaga | taaaccgctctcatgtccaa |
| cagatgctttgaagagcttc | ttgcaaatgtccttccaacc |
| ggtagttggagtatctcttg | cgtcaaaatagttccttccc |
| ggccttagcaagtttcttct | ggaatttacggtcgtgagaa |
| tcttcttccgttcaacaggt | caatgtccgtgcaaatttcg |
| gttgctgatggctcattctt | aatcgtctggtgtgcaaatg |
| tatcctttgagttggctcca | cgtcaacttttccttcatcg |
| gagaagttgtttcgtaccga | gattcgtattctctctgctg |
| aaatgccattgtgacatcgg | gtatgctgtctcaattgctg |
| ccaaccaatccatatcgtct | ggagctcttttagcgttgta |
| cgtttttccgattccgtttc | caggaagaagaacaggcaat |
| gatcttcaactgttggcttg | cgcattctgggaatttgaag |
| acagcattgaaatacctgcg | ggagaactggattactgaga |
| atctccttccacttcctgtt | acggaaggatacttcatcga |
| cagcatccaaaacgagtgta | cggaaaagatagggagaatc |
